# Long-Term Effects of Allogeneic Hematopoietic Stem Cell Transplantation on Systemic Inflammation in Sickle Cell Disease Patients

**DOI:** 10.3389/fimmu.2021.774442

**Published:** 2021-12-09

**Authors:** Júlia Teixeira Cottas de Azevedo, Thalita Cristina de Mello Costa, Keli Cristina Lima, Thiago Trovati Maciel, Patrícia Vianna Bonini Palma, Luiz Guilherme Darrigo-Júnior, Carlos Eduardo Setanni Grecco, Ana Beatriz P. L. Stracieri, Juliana Bernardes Elias, Fabiano Pieroni, Renato Luiz Guerino-Cunha, Ana Cristina Silva Pinto, Gil Cunha De Santis, Dimas Tadeu Covas, Olivier Hermine, Belinda Pinto Simões, Maria Carolina Oliveira, Kelen Cristina Ribeiro Malmegrim

**Affiliations:** ^1^ Center for Cell-Based Therapy, Regional Blood Center of Ribeirão Preto, Ribeirão Preto Medical School, University of São Paulo, Ribeirão Preto, Brazil; ^2^ Graduate Program in Basic and Applied Immunology of the Ribeirão Preto Medicinal School, University of São Paulo, Ribeirão Preto, Brazil; ^3^ Bone Marrow Transplantation and Cellular Therapy Unit, University Hospital, Ribeirão Preto Medical School, University of São Paulo, Ribeirão Preto, Brazil; ^4^ Graduate Program in Bioscience and Biotechnology, School of Pharmaceutical Sciences of Ribeirão Preto, University of São Paulo, Ribeirão Preto, Brazil; ^5^ Institut national de la santé et de la recherche médicale (INSERM) Unité mixte de recherche (UMR) 1163, Centre national de la recherche scientifique (CNRS) Equipe de Recherche Labellisée (ERL) 8254, Laboratory of Cellular and Molecular Mechanisms of Hematological Disorders and Therapeutical Implications, Imagine Institute, Paris, France; ^6^ Imagine Institute, Université Paris Descartes, Sorbonne Paris-Cité et Assistance Publique-Hôpitaux de Paris, Hôpital Necker, Paris, France; ^7^ Department of Pediatrics, Ribeirão Preto Medical School, University of São Paulo, Ribeirão Preto, Brazil; ^8^ Department of Medical Imaging, Hematology, and Clinical Oncology, Ribeirão Preto Medical School, University of São Paulo, Ribeirão Preto, Brazil; ^9^ Department of Internal Medicine, Division of Clinical Immunology, Ribeirão Preto Medical School, University of São Paulo, Ribeirão Preto, Brazil; ^10^ Department of Clinical Analysis, Toxicology and Food Science, School of Pharmaceutical Sciences of Ribeirão Preto, University of São Paulo, Ribeirão Preto, Brazil

**Keywords:** sickle cell disease, chronic inflammation, allogeneic hematopoietic stem cell transplantation, hematological reconstitution, adhesion molecules

## Abstract

Allogeneic hematopoietic stem cell transplantation (allo-HSCT) is the only currently available curative treatment for sickle cell disease (SCD). However, the effects of HSCT on SCD pathophysiology are poorly elucidated. Here, we assessed red blood cell (RBC) adhesiveness, intensity of hemolysis, vascular tone markers and systemic inflammation, in SCD patients treated with allogeneic HSCT. Thirty-two SCD patients were evaluated before and on long-term follow-up after HSCT. Overall survival was 94% with no severe (grade III-IV) graft-*vs*-host disease and a 22% rejection rate (graft failure). Hematological parameters, reticulocyte counts, and levels of lactate dehydrogenase (LDH), endothelin-1 and VCAM-1 normalized in SCD patients post-HSCT. Expression of adhesion molecules on reticulocytes and RBC was lower in patients with sustained engraftment. Levels of IL-18, IL-15 and LDH were higher in patients that developed graft failure. Increased levels of plasma pro-inflammatory cytokines, mainly TNF-α, were found in SCD patients long-term after transplantation. SCD patients with sustained engraftment after allo-HSCT showed decreased reticulocyte counts and adhesiveness, diminished hemolysis, and lower levels of vascular tonus markers. Nevertheless, systemic inflammation persists for at least five years after transplantation, indicating that allo-HSCT does not equally affect all aspects of SCD pathophysiology.

**Graphical Abstract:**
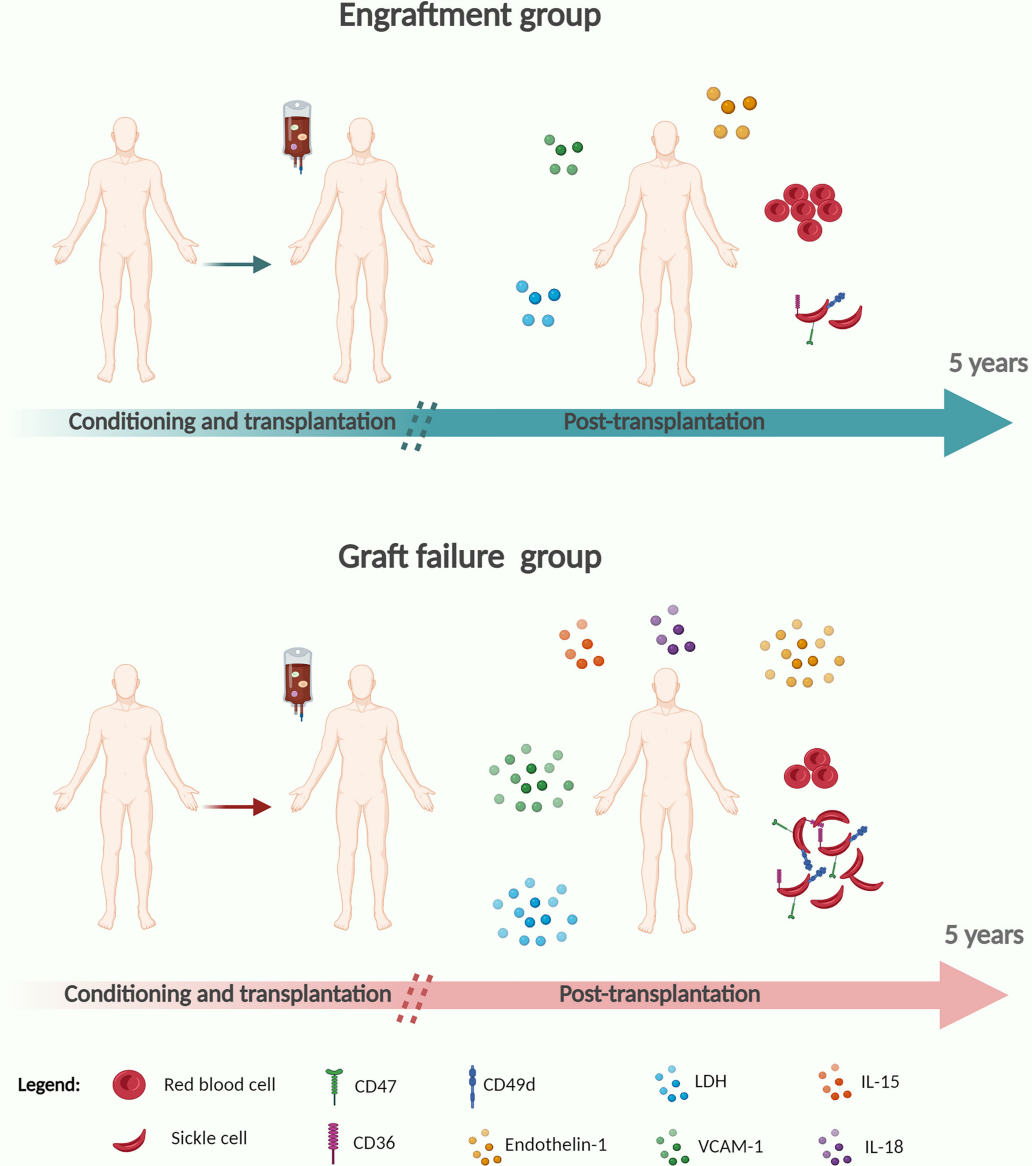
Created with BioRender.com.

## Introduction

Sickle cell disease (SCD) is one of the most prevalent monogenic disease in the world caused by a one point mutation at position 6 of the β-globin gene, which results in abnormal production of hemoglobin S (1–3). SCD pathophysiology is characterized by chronic inflammatory processes, triggered by hemolytic and vaso-occlusive events, which lead to diverse clinical complications, such as chronic hemolytic anemia, splenic sequestration crises, pain, acute chest syndrome, strokes, leg ulcers, retinopathy, dactylitis, priapism, pulmonary hypertension, heart failure, and increased susceptibility to infections (1). The hallmarks of the disease are the vaso-occlusion crises (VOC), severe pain episodes characterized by tissue ischemia and infarction (1, 4), associated with increased levels of soluble VCAM-1, ICAM-1, endothelin-1, IL-6, IL-8, and TNF-α (5–9).

The pathogenesis of SCD is a multifactorial process that comprehends recurrent vaso-occlusions, ischemia-reperfusion injury and oxidative stress (10). In SCD patients, hemoglobin S polymerizes at low oxygen levels, resulting in altered cellular architecture of red blood cells (RBC) and exposure of adhesion molecules on the cell surface, including CD36, CD47, CD49d, and BCAM/Lu (11–14). Sickle red blood cells have a reduced lifespan, leading to accelerated red cell turnover. Circulating reticulocytes, increased in number, express high concentrations of adhesion molecules, contributing to increased adhesiveness of the sickle cells and the occurrence of VOC (11, 15). In addition, sickle cells are more susceptible to lysis and release of free hemoglobin, lactate dehydrogenase (LDH) and arginase in the plasma (16, 17). Free hemoglobin and circulating arginase consume nitric oxide (NO), an important vasodilator (18), further contributing to occlusive crises. Free hemoglobin also releases heme molecules, which in turn activate the immune system and thereby intensify VOC episodes (19, 20).

The vascular endothelium contributes to the inflammatory process in SCD patients. During VOC, damaged endothelial cells trigger the coagulation cascade and induce expression of VCAM-1 (cell-vascular adhesion molecule 1), ICAM-1 (intercellular adhesion molecule 1), P-selectin and E-selectin, which facilitate the adherence of blood cells to the endothelium (17). Endothelial cells produce endothelin-1, not only a potent vasoconstrictor, but also a pro-inflammatory molecule that increases levels of soluble VCAM-1 and ICAM-1 (21, 22), stimulates monocytes to secrete inflammatory cytokines *in vitro* (23) and induces production of superoxide by neutrophils *in vitro* (24, 25).

The management of SCD is a challenge (3, 26, 27) and allogeneic hematopoietic stem cell transplantation (allo-HSCT) is the only currently available curative treatment (28–31). To date, hundreds of SCD patients have already been successfully transplanted worldwide (29, 31). Patients with HLA-identical sibling donors have high overall and event-free survival rates, ranging from 90% to 100% (29, 32). The graft rejection rate (graft failure) varies extensively among different studies (31) and addition of anti-thymocyte globulin (ATG) to the transplant regimen is associated with a decrease in the incidence of graft rejection from 22.6% to 3% (33).

To our knowledge, the effects of allo-HSCT on the pathophysiological processes of SCD have not yet been fully investigated. We would like to understand if systemic inflammation and endothelial dysfunction subside after transplantation and how these events correlate with clinical complications, such as secondary graft failure and graft versus host disease (GVHD). Therefore, in this work we assessed red blood cell adhesiveness, intensity of hemolysis, vascular tone markers and systemic inflammation, in SCD patients treated with allo-HSCT.

## Material, Subjects and Methods

### Patients and Healthy Donors

Thirty-two SCD patients treated with allogeneic HLA-identical sibling HSCT at a single center, between April 2013 and August 2017, had peripheral blood collected for laboratory monitoring at pre-HSCT (baseline) and at 1, 3, 6, 12, 24, 36, 48 and 60 months after allo-HSCT (**Table 1** and **Table S1**). Bone marrow was the stem cell source for all transplants. Except for one, all patients were treated with myeloablative conditioning regimen of fludarabine, busulfan and anti-thymocyte globulin (ATG) (**Tables S1**, **S2**). Methotrexate and cyclosporine A were used as initial GVHD prophylaxis, according to institutional protocol. Eleven patients had cyclosporin-A related toxicity, therefore were administered alternative agents for prophylaxis (sirolimus, mycophenolate mofetil, tacrolimus or corticosteroids). Diagnosis and classification of acute and chronic GVHD were defined according to the Glucksberg modified score and the National Institute of Health 2014 criteria, respectively (34, 35). First line treatment was initiated at diagnosis of GVHD and consisted of corticosteroids.

**Table 1:** Characteristics of 32 SCD patients treated with allogeneic HSCT.

Patient N.	Genotype	Sex/Age (y) at baseline	Race	Baseline treatment	Donor	Conditioning Regimen	Graft function	Death
**SCD01**	HbSS	F/9	White	Chronic transfusion	Sickle cell trait	Myeloablative (BUCY-ATG)	Engraftment	No
**SCD02**	HbSS	M/20	Biracial	Hydroxyurea	Sickle cell trait negative	Myeloablative (BUFLU-ATG)	Engraftment	No
**SCD03**	HbS/β0	F/27	White	Hydroxyurea	Sickle cell trait	Myeloablative (BUFLU-ATG)	Engraftment	No
**SCD04**	HbSS	F/17	White	Hydroxyurea and Chronic transfusion	Sickle cell trait negative	Myeloablative (BUFLU-ATG)	Engraftment	No
**SCD05**	HbSS	F/14	White	Hydroxyurea	Sickle cell trait negative	Myeloablative (BUFLU-ATG)	Engraftment	No
**SCD06**	HbSS	F/10	White	Chronic transfusion	Sickle cell trait	Myeloablative (BUFLU-ATG)	Engraftment	No
**SCD07**	HbS/β0	M/35	Biracial	Chronic transfusion	Sickle cell trait	Myeloablative (BUFLU-ATG)	Engraftment	No
**SCD08**	HbS/β0	F/31	White	Hydroxyurea	Sickle cell trait	Myeloablative (BUFLU-ATG)	Poor graft Function	Yes
**SCD09**	HbSS	M/24	Biracial	Hydroxyurea and Chronic transfusion	Sickle cell trait negative	Myeloablative (BUFLU-ATG)	Engraftment	No
**SCD10**	HbS/β0	M/13	White	Chronic transfusion	Sickle cell trait negative	Myeloablative (BUFLU-ATG)	Failure	No
**SCD11**	HbS/β0	F/30	White	Chronic transfusion	Sickle cell trait	Myeloablative (BUFLU-ATG)	Engraftment	No
**SCD12**	HbS/β0	M/25	Biracial	Hydroxyurea	Sickle cell trait negative	Myeloablative (BUFLU-ATG)	Failure	No
**SCD13**	HbS/HbC	M/7	Biracial	Hydroxyurea and Chronic transfusion	Sickle cell trait	Myeloablative (BUFLU-ATG)	Engraftment	No
**SCD14**	HbSS	M/18	White	Hydroxyurea and Chronic transfusion	Sickle cell trait	Myeloablative (BUFLU-ATG)	Engraftment	No
**SCD15**	HbSS	F/14	White	Hydroxyurea	Sickle cell trait	Myeloablative (BUFLU-ATG)	Failure	No
**SCD16**	HbSS	F/23	Biracial	Chronic transfusion	Sickle cell trait negative	Myeloablative (BUFLU-ATG)	Engraftment	No
**SCD17**	HbSS	F/19	White	Hydroxyurea	Sickle cell trait	Myeloablative (BUFLU-ATG)	Engraftment	No
**SCD18**	HbSS	M/14	Biracial	Hydroxyurea and Chronic transfusion	Sickle cell trait negative	Myeloablative (BUFLU-ATG)	Engraftment	No
**SCD19**	HbSS	F/10	White	Chronic transfusion	Sickle cell trait	Myeloablative (BUFLU-ATG)	Engraftment	No
**SCD20**	HbSS	M/11	Biracial	Hydroxyurea and Chronic transfusion	Sickle cell trait	Myeloablative (BUFLU-ATG)	Engraftment	No
**SCD21**	HbSS	M/10	White	Chronic transfusion	Sickle cell trait negative	Myeloablative (BUFLU-ATG)	Failure	No
**SCD22**	HbSS	M/30	Biracial	*	Sickle cell trait	Myeloablative (BUFLU-ATG)	Engraftment	No
**SCD23**	HbS/HbC	M/26	White	Hydroxyurea	Sickle cell trait	Myeloablative (BUFLU-ATG)	Engraftment	No
**SCD24**	HbS/β0	M/16	White	Hydroxyurea	Sickle cell trait negative	Myeloablative (BUFLU-ATG)	Engraftment	No
**SCD25**	HbSS	M/12	White	Chronic transfusion	Sickle cell trait	Myeloablative (BUFLU-ATG)	Engraftment	No
**SCD26**	HbSS	M/20	Biracial	Hydroxyurea	Sickle cell trait	Myeloablative (BUFLU-ATG)	Engraftment	No
**SCD27**	HbSS	M/7	White	Chronic transfusion	Sickle cell trait	Myeloablative (BUFLU-ATG)	Engraftment	No
**SCD28**	HbSS	F/33	Black	Hydroxyurea	Sickle cell trait	Myeloablative (BUFLU-ATG)	Engraftment	No
**SCD29**	HbSS	M/12	White	Hydroxyurea	Sickle cell trait	Myeloablative (BUFLU-ATG)	Engraftment	No
**SCD30**	HbSS	F/32	White	Hydroxyurea	Sickle cell trait negative	Myeloablative (BUFLU-ATG)	Failure	Yes
**SCD31**	HbSS	F/13	White	Simple and Chronic transfusion	Sickle cell trait	Myeloablative (BUFLU-ATG)	Failure	No
**SCD32**	HbSS	F/12	Biracial	Hydroxyurea and Chronic transfusion	Sickle cell trait	Myeloablative (BUFLU-ATG)	Engraftment	No

*Not available.

Graft failure was classified as primary, when patients never achieved an absolute neutrophil count > 0.5 X 10^9^/L, or as secondary, when patients lost the donor chimera, identified through qualitative variable number tandem repeats (VNTR) analysis (36).

The median (range) of follow-up was 34 (4–52) months, seven (21.9%) for the SCD-patients who had graft failure. Fifteen (46,9%) patients developed non-life threatening aGVHD (grade I or II) and six (18,6%) cGVHD (**Table S1**). All patients who developed GVHD (acute or chronic) were successfully treated. None of the patients presented primary graft failure.

We retrospectively clustered patients into engraftment group – 25 patients with sustained engraftment after transplantation, with complete or mixed donor chimera by qualitative VNTR analysis; and graft failure group – 7 patients who presented secondary graft failure (**Table S3**). Groups were similar for age, gender, race, and conditioning regimen intensity (**Table S4**). We also clustered patients in aGVHD - patients who developed acute GVHD and non-GVHD - patients who did not develop acute or chronic GVHD. Three patients who developed only chronic GVHD were not included.

Peripheral blood samples from 19 healthy donors (controls) were collected and used for comparisons. The Institutional Ethics Committee approved all protocols (processes number 3551/2002 and 2479/2015). Patients and controls (or a legally authorized representative) signed informed consent forms before study enrollment.

### Blood Counts and Hemoglobin S Quantification

Red cell counts, and hemoglobin concentrations were measured on a KX-21N automatic analyzer (Sysmex America, Inc., USA). Hemoglobin S was quantified by high-performance liquid chromatography (HPLC) technique using a Variant II (Bio-Rad Laboratories Inc., California, USA), according to manufacturer’s recommendations.

### Hematological Characterization

Reticulocyte frequency and expression of adhesion molecules (CD36, CD47 and CD49d) on reticulocytes were assessed by flow cytometry. Cells were analyzed using FACSCalibur flow cytometer (Becton-Dickinson, San Diego, CA, USA) and FlowJo software (FLOWJO LLC, Oregon, USA). For the evaluation of reticulocyte percentage, monoclonal antibodies CD36-PE, CD47-PE and CD49d-PerCP-Cy5.5 (Becton-Dickinson, San Diego, CA, USA) and Thiazole Orange (Sigma, St. Louis, Missouri, USA) were used. Whole peripheral blood (5 μl) was incubated with 1 mL of Thiazole Orange or with 1 ml of PBS 1x (control) for 30 minutes in the dark, at room temperature. For the analysis of adhesion molecules, whole blood was centrifuged at 270 g for 3 minutes, plasma was discarded, and the red cell solution was washed with PBS1x three times, diluted 200-fold (10 μL red cell solution plus 1990 μL PBS 1x) and used for labeling. Aliquots of diluted red blood cells (100μL) were incubated with 5 μL of monoclonal antibodies or control isotypes for 15 minutes in the dark, at room temperature. Then, 1mL of Thiazole Orange (or PBS 1x in the control tubes) was added and incubated for 30 minutes in the dark, at room temperature. Results were given as percentages of reticulocytes, Thiazole+CD36+ cells, Thiazole+CD49d+ and Thiazole+CD47+. Detailed gate strategy is available in **Figure S1**.

### Plasma Soluble Adhesion Molecules Dosage

Plasma adhesion molecules were determined by multiplex assays (Magnetic Luminex Assay, R&D System Inc., Minnesota, USA or Bio-Plex, Bio-Rad Laboratories Inc., California, USA), according to manufacturers’ recommendations.

### Plasma Inflammatory Mediators Dosage

TGF-β was measured by Cytometric Bead Array flex (CBA; Becton-Dickinson, San Jose, CA, EUA) and other inflammatory mediators were determined by multiplex assays (Magnetic Luminex Assay, R&D System Inc., Minnesota, USA or Bio-Plex, Bio-Rad Laboratories Inc., California, USA), according to manufacturers’ recommendations.

### Nitric Oxide Dosage

Serum quantification of products derived from nitric oxide metabolism (nitrite and nitrate) was performed by colorimetric assay (GREISS reaction) (37).

### Heme and Lactate Dehydrogenase (LDH) Dosage

Serum heme levels were analyzed by Heme Assay Kit (Sigma-Aldrich, Steinhein, Germany) and LDH levels were analyzed by optimized UV method (Wiener lab, Rosario, AR), according to manufacturer’s recommendations.

### Data Analysis

A linear regression mixed model, composed of random and fixed effects, was used to analyze data from hematological reconstitution and plasma marker levels. For variable frequency, the data underwent logarithmic transformation. This model allows multiple longitudinal observations per individual across a baseline period and subsequent time points after transplantation. Besides, this model applies to the analysis of data in which responses are grouped (more than one measure to the same individual), when the assumption of independence among observations in the same group is not adequate. The fixed effects were groups and periods. The random effects were associated with patients since it was necessary to control correlations among repeated measures. For variable frequency, we used a logarithmic transformation to fit the data to the proposed model. The analyses of each variable were controlled by patient’s age. Data analysis was performed using SAS^®^9.0 statistical software (SAS Institute Inc., Cary, NC, USA). Receiver-operating characteristic (ROC) curves were calculated to identify potential markers of graft failure and area under the curves (AUC) ≥ 0.7 were considered. Significance was set at P<.05 and n≥3. Statistical significance was set at p < 0.05.

## Results

This study included 32 SCD patients who were treated with allogeneic HSCT. Patients were followed-up for a mean (standard deviation) time of 30 (13) months, and clustered according to post-transplant chimerism determined by VNTR. Two patients from the graft failure group died at 7 and 49 months after transplantation, both due to infectious complications.

### Normalization of Hematological Parameters After Transplantation

**Figure 1:**
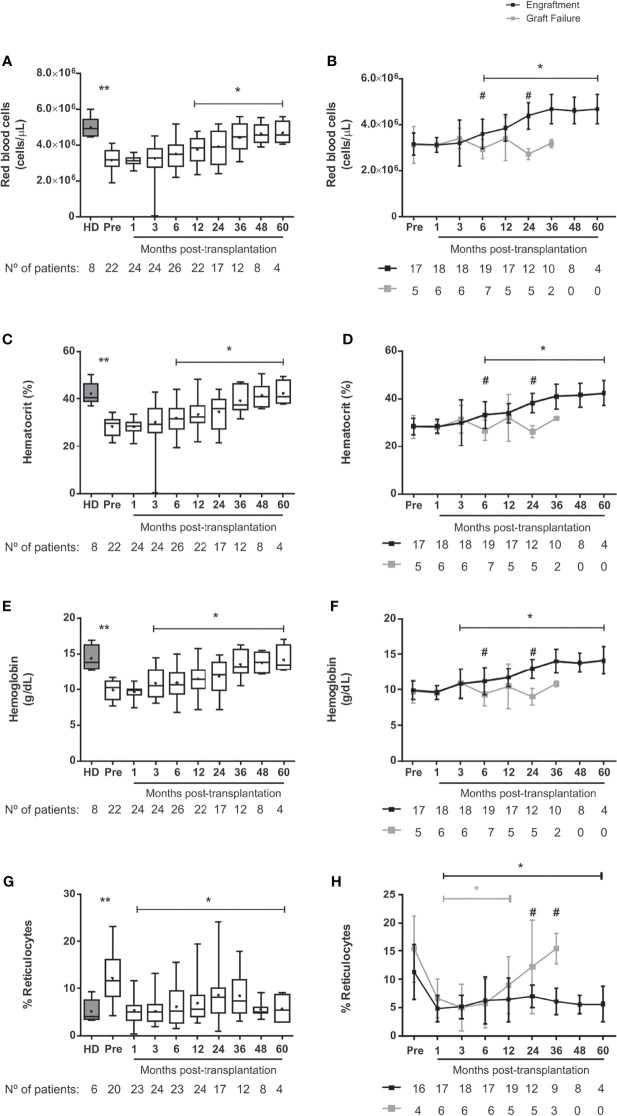
Erythropoiesis in SCD-patients following allogeneic HSCT. **(A)** red blood cell counts in transplanted patients, **(B)** red blood cell counts in patients divided according to graft function, **(C)** hematocrit in transplanted patients, **(D)** hematocrit in patients divided according to graft function, **(E)** hemoglobin in transplanted patients, **(F)** hemoglobin in patients divided according to graft function, **(G)** reticulocytes in transplanted patients and **(H)** reticulocytes in patients divided according to graft function. Black lines represent the engraftment group and gray lines represent the graft failure group, + indicate the means. Statistical analysis was performed using a model of multiple regression of mixed effects. *Statistical difference between pre- and post-transplantation time points in the overall group of patients **(A, C, E, G)** or in each group **(B, D, F, H)** (P < 0.05); **Statistical difference between healthy donors and pre-transplant (P < 0.05). ^#^Statistical difference between engraftment group and graft failure group (P < 0.05); HD, healthy donor; Pre, pre-transplantation period.

At baseline, patients presented low numbers of red blood cells, hematocrit and hemoglobin levels and increased reticulocyte counts when compared to healthy donors (**Figure 1**). Early after HSCT, there was an increase in the number of red blood cells, hematocrit and hemoglobin levels, while the reticulocyte counts decreased. When patients were clustered according to engraftment, the graft failure group had lower red blood cell counts, hematocrit, and hemoglobin levels at 6 and 24 months after HSCT and higher reticulocyte counts at 24 and 36 months compared to the engraftment group (**Figures 1B** **, D, F, H**). **Figure S2** shows hemoglobin S levels at different time points in patients clustered according to post-transplantation engraftment.

### Adhesion Molecules Decrease in Red Blood Cells, but Not in the Peripheral Blood

**Figure 2:**
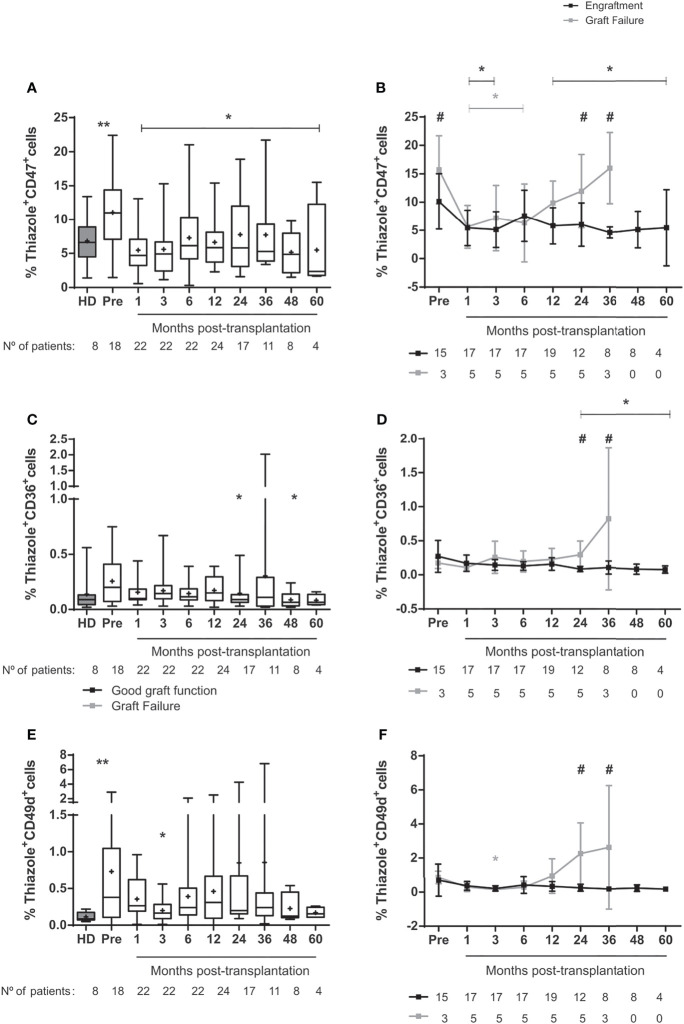
Expression of adhesion molecules in reticulocytes from SCD-patients treated with allogeneic HSCT. **(A)** percentage of reticulocytes expressing CD47 in the overall group of transplanted patients, **(B)** percentage of reticulocytes expressing CD47 in patients divided according to graft function, **(C)** percentage of reticulocytes expressing CD36 in transplanted patients, **(D)** percentage of reticulocytes expressing CD36 in patients divided according to graft function, **(E)** percentage of reticulocytes expressing CD49d in transplanted patients and **(F)** percentage of reticulocytes expressing CD49d in patients divided according to graft function. Black lines represent the engraftment group and gray lines represent the graft failure group, + indicate the means. Statistical analysis was performed using a model of multiple regression of mixed effects. *Statistical difference between pre- and post-transplantation time points in the overall group of patients **(A, C, E)** or in each group **(B, D, F)** (P < 0.05); **Statistical difference between healthy donors and pre-transplantation (P < 0.05). ^#^Statistical difference between engraftment group and graft failure group (P < 0.05); HD, healthy donor; Pre, pre-transplantation period.

To evaluate the impact of HSCT on cellular adhesiveness, we measured the expression of adhesion molecules on reticulocytes and mature RBC in SCD patients before and after transplantation and in healthy individuals. The frequency of reticulocytes expressing CD47 was higher in SCD patients at baseline compared with healthy donors (**Figure 2A**). This frequency decreased shortly after the procedure and remained low up to 60 months after transplantation. Reticulocytes expressing CD49d were more frequent at baseline than in healthy donors but decreased at 3 months after transplantation (**Figure 2E**). CD36 expression on SCD reticulocytes at baseline was not different from healthy controls and decreased at 24 and 48 months after transplantation (**Figure 2C**).

In the engraftment group, frequencies of reticulocytes expressing CD47 remained lower than baseline throughout most of the post-transplantation follow-up (**Figure 2B**) and the expression of CD36 on reticulocytes diminished from 24-60 months post-transplantation (**Figure 2D**). At 24-36 months after transplantation, the expression of adhesion molecules CD47, CD36 and CD49d were higher in the graft failure group compared with the engraftment group (**Figures 2B** **, D, F**).

**Figure 3:**
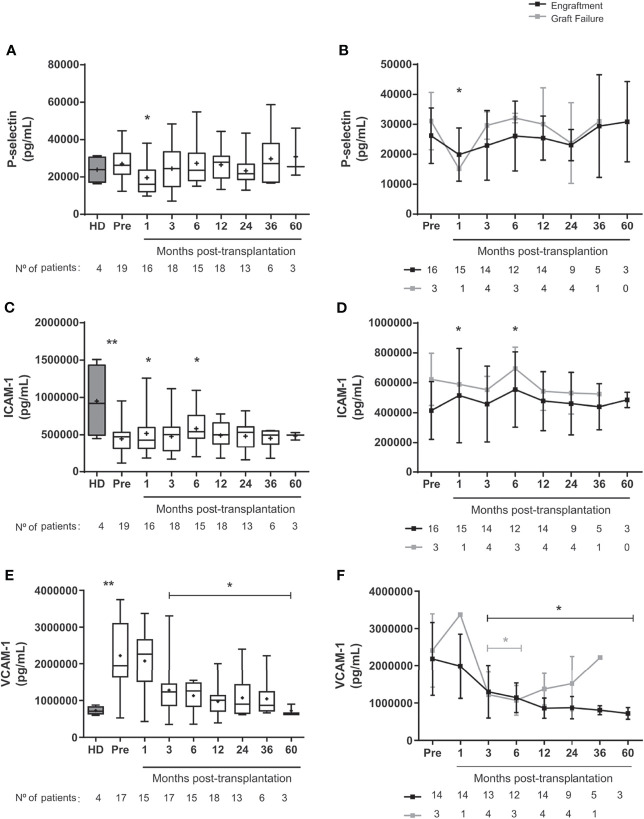
Levels of soluble adhesion molecules in SCD patients following allogeneic HSCT. Concentration of **(A)** P-selectin in the overall group of transplanted patients, **(B)** P-selectin in patients divided according to graft function, **(C)** ICAM-1 in the overall group of transplanted patients, **(D)** ICAM-1 in patients divided according to graft function, **(E)** VCAM-1 in the overall group of transplanted patients and **(F)** VCAM-1 in patients divided according to graft function. Black line representing the engraftment group and gray line representing the graft failure group, + indicate the means. Statistical analysis was performed using a model of multiple regression of mixed effects. *Statistical difference between pre- and post-transplantation time points in the overall group of patients **(A, C, E)** or in each group **(B, D, F)** (P <0.05); **Statistical difference between healthy donors and pre-transplantation (P < 0.05); HD, healthy donor; Pre, pre-transplantation period.

In the analyses of mature cells, we observed increased frequencies of RBC expressing CD47 (**Figure S3A**), mainly in the engraftment group (**Figure S3B**). RBC expressing CD36 decreased 36-48 months post-transplantation in patients with successful engraftment (**Figures S3C, D**), while there were no changes in CD49d expression in mature RBC (**Figures S3E, F**). The expression of adhesion molecules CD36 and CD49d was increased in the graft failure group compared to the engraftment group at 36 months (**Figures 3D** **, F**).

In patients with good engraftment, the plasma concentration of P-selectin decreased at one month (**Figures 3A** **, B**), while ICAM-1 levels increased at one- and 6-months post-transplantation (**Figures 3C** **, D**). Before transplantation, the concentration of ICAM-1 was higher in healthy donors than in SCD patients (**Figure 3C**). Conversely, VCAM-1 levels were higher in SCD than in controls and decreased up to 5 years after HSCT compared to baseline (**Figures 3E** **, F**). We did not find sustained changes on reticulocytes counts, the expression of adhesion molecules on reticulocytes and mature RBC, and the soluble levels of adhesion markers when patients were clustered according to GVHD occurrence (**Figures S4**, **S5**).

### Hemolysis Markers Improve After HSCT

**Figure 4:**
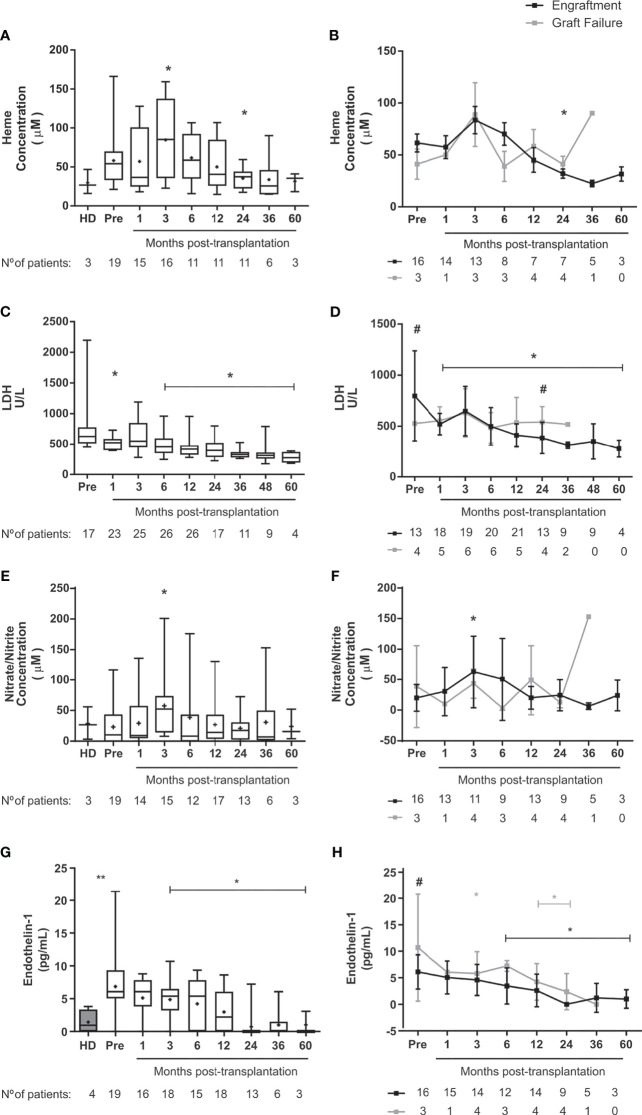
Markers of hemolysis and muscular tone in SCD patients following allogeneic HSCT. Concentration of **(A)** Heme in the overall group of transplanted patients, **(B)** Heme in patients divided according to graft function, **(C)** LDH in the overall group of transplanted patients, **(D)** LDH in patients divided according to graft function. **(E)** Nitrite and Nitrate in the overall group of transplanted patients, **(F)** Nitrite and Nitrate in patients divided according to graft function, **(G)** Endothelin-1 in the overall group of transplanted patients, **(H)** Endothelin-1 in patients divided according to graft function. Black line representing the engraftment group and gray line representing the graft failure group, + indicate the means. Statistical analysis was performed using a model of multiple regression of mixed effects. *Statistical difference between pre- and post-transplantation time points in the overall group of patients **(A, C, E, G)** or in each group **(B, D, F, H)** (P <0.05); **Statistical difference between healthy donors and pre-transplantation (P < 0.05). ^#^Statistical difference between engraftment group and graft failure group (P < 0.05); HD, healthy donor; Pre, pre-transplantation period.

There was a brief increase of heme concentrations at 3 months and a transitory decrease at 24 months after transplantation in the overall population (**Figures 4A** **, B**). Conversely, LDH levels decreased up to 5 years after transplantation in the engraftment group compared to baseline (**Figures 4C** **, D**).

### Vascular Tone Markers Are Decreased in SCD Patients Following HSCT

While there was transient increase of NO metabolites at 3 months after transplantation (**Figures 4E** **, F**), we detected sustained decrease of endothelin-1 from 6 months until last follow-up post-HSCT in patients with successful engraftment (**Figures 4G** **, H**). Of note, patients in the graft failure group had increased levels of endothelin-1 already at baseline (**Figures 4H**). We did not find sustained changes on NO metabolites and endothelin-1 levels when patients were clustered according to occurrence of acute GVHD (**Figure S5**).

### Changes in Inflammatory Mediators After HSCT

**Figure 5:**
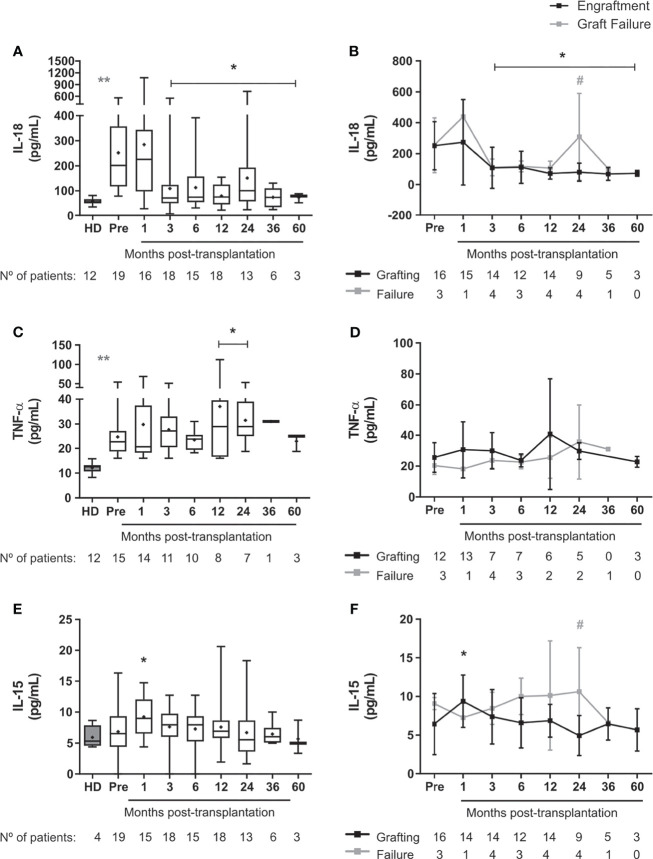
Levels of pro-inflammatory cytokines in SCD patients following allogeneic HSCT. Concentration of **(A)** IL-18 in the overall group of transplanted patients, **(B)** IL18 in patients divided according to graft function, **(C)** TNF-α in the overall group of transplanted patients, **(D)** TNF-α in patients divided according to graft function, **(E)** IL-15 in the overall group of transplanted patients, **(F)** IL-15 in patients divided according to graft function. Black line representing the engraftment group and gray line representing the graft failure group, + indicate the means. Statistical analysis was performed using a model of multiple regression of mixed effects. *Statistical difference between pre- and post-transplantation time points in the overall group of patients **(A, C, E)** or in each group **(B, D, F)** (P < 0.05); **Statistical difference between healthy donors and pre-transplantation (P < 0.05). ^#^Statistical difference between engraftment group and graft failure group (P < 0.05); HD, healthy donor; Pre, pre-transplantation period.

Levels of TNF-α and IL-18 were higher in SCD patients at baseline compared to healthy controls (**Figures 5A, C**). Levels of IL-18 decreased until 5-year post-transplantation in engrafted patients, but levels of TNF-α increased at 1-2 years after HSCT (**Figures 5B, D**). Following transplantation, we did not observe changes in plasma levels of IL-8, IL-21, IL-22, and IL-33. TGF-β levels were reduced for 3 months after transplantation in patients with good engraftment (**Figure S6**). The concentrations of IL-15 and IL-18 increased at 2 years after HSCT in patients who underwent graft failure (**Figures 5A, B, E** **, F**). Interestingly, patients who developed aGVHD presented increased levels of HGF at 1 month after transplantation as well as decreased levels of TGF-β at 36 months after allo-HSCT (**Figure S7**). Other cytokines were not detected in SCD patients enrolled in this study (**Table S5**).

## Discussion

Hematological parameters normalize in SCD patients successfully engrafted following allogeneic HSCT (29, 33, 38–43). Indeed, here we show that HSCT modifies key elements associated with SCD pathogenesis.

Sickle-cell patients have increased numbers of reticulocytes, with high expression of adhesion molecules such as CD49d, CD36 and Lu-BCAM (11). Accordingly, we detected increased percentages of reticulocytes expressing CD49d in our SCD patients at baseline, when compared to controls. After HSCT, patients with successful engraftment had lower frequencies of reticulocytes expressing CD49d and CD36 than patients with graft failure, suggesting that HSCT substitutes highly adherent abnormal cells by normal adherent cells. A similar mechanism has been described in sickle cell patients under treatment with hydroxyurea, with decreased expression of adhesion molecules, such as CD36 and CD49d, on reticulocytes (11, 44).

Our SCD patients also had higher percentage of reticulocytes expressing CD47 at baseline when compared to the healthy donors. These percentages were reduced after transplantation in patients with good engraftment. Surprisingly, however, the expression of CD47 was increased in mature RBC of SCD after transplantation. CD47 is involved with increased cell adhesion in SCD patients as part of disease mechanism (45, 46). CD47 expressed on SCD reticulocytes is able to bind to thrombospondin, resulting in VLA-4 activation, thereby increasing their adhesion properties (45, 46). CD47 also plays an important role in regulating the clearance of senescent RBC by macrophages, as it binds to macrophage inhibitory receptors (SIRPα) and prevents phagocytosis (47, 48). This may explain the greater expression of this molecule in mature RBC after transplantation.

Plasma concentrations of P-selectin, VCAM-1 and ICAM-1 are increased in SCD patients, a phenomenon associated with severe clinical symptoms and tissue damage (6, 7, 9, 49, 50). In our SCD patients, however, baseline concentrations of P-selectin were similar to those from healthy controls, whereas concentrations of ICAM-1 were lower than controls. VCAM-1 plasma concentrations, conversely, were higher than controls at baseline and decreased significantly after HSCT, especially in patients with good engraftment. The divergences between ICAM-1 and P-selectin levels measured in our patients and those reported by the literature may be due to number of evaluated patients and should be further evaluated in future studies.

Lactate dehydrogenase (LDH) levels were reduced up to 5 years after HSCT in patients with good engraftment, which is in accordance with the successful hematological recovery observed in our patients. Although LDH has been established as a biomarker of hemolysis and is associated with severe clinical symptoms of SCD, such as priapism, leg ulcers, pulmonary hypertension and death (16), it also reflects tissue damage, which is involved in the pathogenesis of the disease (51). Thus, the decreasing levels of LDH may reflect not only an improvement of hemolysis, but also from reduction in tissue damage and ischemia.

In our patients, IL-15 and IL-18 levels remained elevated in SCD patients who experienced graft failure, in contrast with those with sustained engraftment. It is not clear if the quantitative changes of these cytokines are cause or consequence of graft failure since autologous recovery of abnormal hematopoiesis after graft failure may also resume the pre-transplantation inflammatory status. Organ transplantation studies suggest that IL-15 mediates allograft rejection mainly by NK activation (52) and IL-18 has been associated with poor allograft function (53). Conversely, IL-18 is associated with hemolysis and endothelial activation in SCD patients, suggesting that this cytokine may contribute to vaso-occlusion pathophysiology (54). Interestingly, Cerqueira and collaborators (54) demonstrated a positive correlation between IL-18 and uric acid plasma levels in SCD patients. Uric acid is a damage-associated molecular pattern (DAMP) and may trigger the NLRP3 inflammasome pathway, resulting in secretion of IL-1β and IL-18 (54). In our study, however, we were not able to find an association between uric acid levels and clinical outcomes, possibly because of the administration of cyclosporine A to the patients, a known urate retention agent (data not shown). Nevertheless, we believe that NLRP3 inflammasome activation could be a possible underlying mechanism of IL-18 production in SCD patients that underwent graft failure after HSCT. The participation of NLRP3 inflammasome and possible triggers in graft failure should be more specifically investigated in future studies.

Heme levels in our SCD patients were not higher than control levels at baseline, possibly because patients were clinically stable immediately before transplantation, not presenting sickle cell crises or severe anemia. Shortly after HSCT, procedure-related events may have contributed to transient increase in heme levels, since toxicity from the conditioning regimen, infections, blood component transfusions with high levels of free heme, ABO incompatibility, adverse effects from drugs and graft versus host disease (GVHD) may activate the endothelium and the immune system (55–57). Later on follow-up, heme levels declined and resumed normal levels, which can be considered another indication of favorable outcomes after HSCT. Heme is critical for endothelial and inflammatory activation in SCD (17, 19, 58, 59). Indeed, in patients who presented graft failure, heme levels increased after 2 years, due to recurrence of hemolytic anemia and, probably, of the endothelial dysfunction, as supported by the VCAM-1 levels. Furthermore, heme has also been described as an activator molecule of NLRP3 inflammasome (60), which may explain the increased levels of IL-18 observed in SCD patients at pre-transplantation and in those who underwent graft failure.

Recent reports have associated high levels of pre-transplant IL-18 with non-relapse and overall mortalities (61) and delayed platelet recovery in transplanted patients (62). We speculate that the mixed chimerism achieved after allogeneic HSCT, marked by 20-40% HbS in recipient sickle red blood cells, may lead to release of endogenous ligands that trigger NLRP3 inflammasome activation (such as heme, uric acid). This activation may initiate the secretion of mature forms of IL-18 from endothelial cells to promote further inflammatory processes and oxidative stress in the endothelium. Administration of immunotherapeutic agents (such as drugs targeting NLRP3 inflammasome pathway) may diminish endothelial activation and, as consequence, decrease systemic inflammation in SCD patients after HSCT. Future studies addressing the role of NLRP3 inflammasome activation in graft failure may shed light on HSCT protocol improvements for SCD patients.

Finally, considering the pivotal role of endothelial function in clinical events associated with SCD, we evaluated mediators of vascular tonus associated with VOC in SCD patients (12, 63, 64). Endothelin-1 levels were increased at baseline, when compared to healthy controls, and decreased after HSCT, especially in patients with good engraftment. Baseline endothelin-1 plasma levels in patients who later developed graft failure were already higher than in patients with good engraftment after HSCT. However, ROC curve analyses failed to demonstrate associations between levels of endothelin-1 at baseline and transplant outcomes, probably due to reduced number of patients analyzed. A potential predictive role of endothelin-1 should be tested in a larger cohort of SCD patients treated with HSCT.

Notably, the presence of acute GVHD in our cohort of patients seem not to affect the assessment of systemic inflammatory parameters, except for the levels of HGF and TGF-β. The levels of HGF were increased early post-transplant in SCD patients with aGVHD, which is in accordance with the literature that have described HGF as an important biomarker for aGVHD occurrence (65, 66).

We acknowledge here some limitations of this work. First, this study lacked a treatment control and a non-SCD transplanted control group of patients for comparisons. We would need an age-matched group of patients with other hematological reconstitution, such as aplastic anemia, also treated with allo-HSCT, for demonstrating whether the sustained systemic inflammation after is specific of SCD. Second, the number of overall patients evaluated in this study is small for biomarkers and multivariate analyses. Thereby, statistical analyses are difficulted and there is a potential risk of bias, which should be acknowledged. Furthermore, these data need to be validated in other carefully designed controlled clinical studies and/or in other cohorts of patients.

Here, we showed that systemic inflammation persists in SCD patients long-term after allo-HSCT, evidenced by altered levels of plasma pro-inflammatory cytokines (mainly TNF-α) adhesion molecules (P-selectin and ICAM-1), indicating that transplantation does not equally affect all aspects of SCD pathogenesis. At 24 months or later after transplantation, TNF-α levels were persistently high. In SCD patients who underwent graft failure, IL-18 and IL-15 levels were significantly increased at 24 months after allo-HSCT. Following transplantation, we observed increased production of healthy mature erythrocytes, decreased circulating reticulocyte counts and adhesiveness, decreased hemolysis, and improved vascular tone markers in SCD patients with successful engraftment.

## Data Availability Statement

The original contributions presented in the study are included in the article/**Supplementary Material**. Further inquiries can be directed to the corresponding author.

## Ethics Statement

The studies involving human participants were reviewed and approved by Ethics Committee of the Ribeirão Preto Medical School, University of São Paulo. Written informed consent to participate in this study was provided by the participants’ legal guardian/next of kin.

## Author Contributions

Study concept and design: KM, BS, and MO. Study supervision: KM, BS, and MO. Acquisition of clinical data: TC, LD-J, CS, AS, JE, FP, RG-C, AP, GS, BS, and MO. Acquisition of laboratorial data: JA, TM, KL, and PP. Analysis and interpretation of data: JA, TC, KM, and MO. Administrative, technical, or material support: DC and OH. Obtained funding: KM, BS, MO, DC, and OH. Drafting of the manuscript: JA, TC, KM, and MO. Critical revision of the manuscript: JA, TC, TM, KL, PP, TC, LD-J, CS, AS, JE, FP, RG-C, AP, GS, DC, OH, BS, and MO. All authors contributed to the article and approved the submitted version.

## Funding

This work was supported by two Brazilian foundations: São Paulo Research Foundation (Center for Cell-Based Research, CTC-CEPID-FAPESP, grant #2013/08135-2; grant #2014/00088-8, grant #2016/22330-0) and the Coordination for the Improvement of Higher Education Personnel (CAPES; Finance Code 001). This work was supported by State funding from the French National Research Agency under “Investissements d’Avenir” program (ANR-10-IAHU-01) and ANR-16-CE14-0024-03 «Tiebet ». JTC de Azevedo was supported by a labex GR-Ex fellowship. The labex GR-Ex, reference ANR-11-LABX-0051, is funded by the program “Investissements d’avenir” of the French National Research Agency, reference ANR-11-IDEX-0005-02.

## Conflict of Interest

The authors declare that the research was conducted in the absence of any commercial or financial relationships that could be construed as a potential conflict of interest.

## Publisher’s Note

All claims expressed in this article are solely those of the authors and do not necessarily represent those of their affiliated organizations, or those of the publisher, the editors and the reviewers. Any product that may be evaluated in this article, or claim that may be made by its manufacturer, is not guaranteed or endorsed by the publisher.
