# Supplementary material for: Long-Term Effects of Allogeneic Hematopoietic Stem Cell Transplantation on Systemic Inflammation in Sickle Cell Disease Patients
Source: Front Immunol. 2021 Dec 9;12:774442. doi: 10.3389/fimmu.2021.774442 (PMC8696202; doi:10.3389/fimmu.2021.774442)
Supplement: Supplementary file 6 [file DataSheet_6.docx]

**Supplementary Material**

**Title:** Long-term effects of allogeneic hematopoietic cell transplantation on systemic inflammation in sickle cell disease patients

**Authors:** Júlia Teixeira Cottas de Azevedo, Thalita Cristina de Mello Costa, Keli Lima, Thiago Trovati Maciel, Patrícia V. B. Palma, Luiz Guilherme Darrigo Júnior, Carlos Eduardo Setanni Grecco, Juliana Bernardes Elias, Ana Beatriz. P. L. Stracieri, Fabiano Pieroni, Renato Cunha, Ana Cristina Silva Pinto, Gil Cunha De Santis, Dimas Tadeu Covas, Olivier Hermine, Belinda P. Simões, Maria Carolina Oliveira and Kelen C. R. Malmegrim

**Summary**

| **Supplemental Figures** ......................................................................................................................... | | 2 |
| --- | --- | --- |
|  | |  |
| **Figure S1** | Gate strategy for analysis of adhesion molecules expression in red blood cells………… | 2 |
| **Figure S2** | Hemoglobin S level in SCD patients following allogeneic HSCT……………………….. | 3 |
| **Figure S3** | Expression of adhesion molecules in mature RBC from SCD-patients treated with allogeneic HSCT ………………………………………………………………………… | 4 |
| **Figure S4** | Expression of adhesion molecules in reticulocytes and mature RBC from SCD-patients treated with allogeneic HSCT …………………………………………………………… | 5 |
| **Figure S5** | Levels of soluble adhesion molecules and markers of hemolysis and muscular tone in SCD patients following allogeneic HSCT ………………………………………………. | 6 |
| **Figure S6** | Levels of inflammatory mediators in SCD patients following allogeneic HSCT.............. | 7 |
| **Figure S7** | Levels of inflammatory mediators in SCD patients following allogeneic HSCT ………. | 8 |
|  | |  |
| **Supplemental Tables**.............................................................................................................................. | | 9 |
|  | |  |
| **Table S1** | Patient characteristics and clinical outcomes of 32 SCD patients treated with allogeneic HSCT ……………………………………………………………………………………. | 9 |
| **Table S2** | Patients retrospectively clustered in graft failure and engraftment groups ……………… | 12 |
| **Table S3** | Qualitative chimerism of SCD patients after allogeneic HSCT …………………………. | 14 |
| **Table S4** | Intergroup statistical analyses regarding age, gender and race of retrospectively divided groups …………………………………………………………………………………… | 15 |
| **Table S5** | Soluble mediators of inflammatory response in SCD patients submitted to allogeneic HSCT ……………………………………………………………………………………. | 16 |
| **Table S6** | ROC curves analysis of endothelin-1 levels before transplantation …………………….. | 17 |
| **Excel Files** | Results of the Statistical analyses ……………………………………………………….. | 18 |

**Supplemental Figures**

**
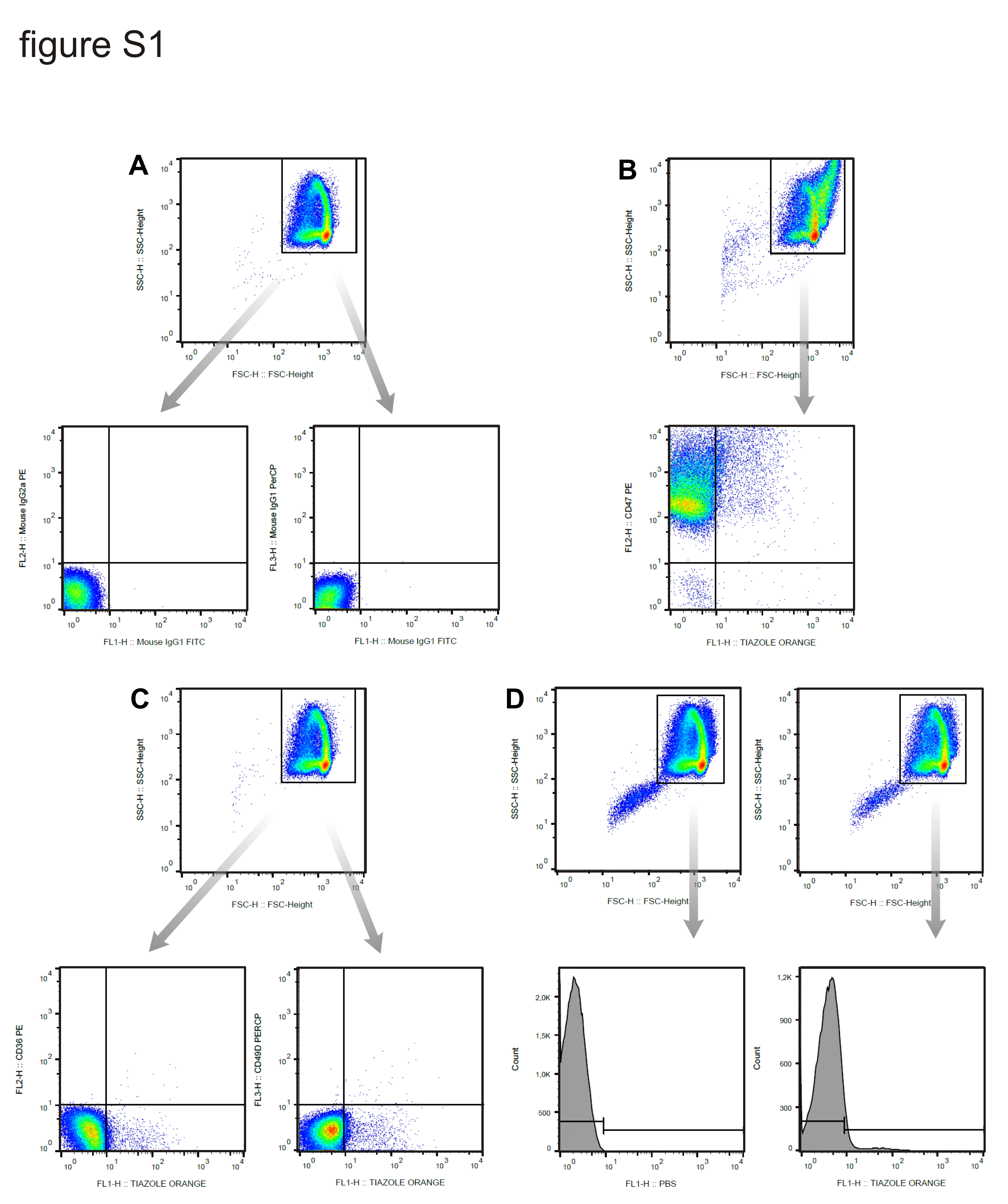
**

**Figure S1 - Gate strategy for analysis of adhesion molecules expression in red blood cells.** For analyses of adhesion molecules expression in red blood cell (RBC), the cells were acquired from red blood cells solution and the gate was drawn in RBC based on size (FSC) and granularity (SSC) parameters. (**A**) The fluorescence FL1 *versus* FL2 and FL1 *versus* FL3 were represented by dot plots established from isotype control. It was represented an example of (**B**) CD47 and Thiazole labeling (FL1 *versus* FL2), (**C**) CD36 and Thiazole labeling (FL1 *versus* FL2) and CD49d and Thiazole labeling (FL1 *versus* FL3). (**D**) For reticulocytes analyses, the cells were acquired from peripheral blood and the gate was designed RBC population based on size (FSC) and granularity (SSC) parameters. The fluorescence (FL1, Thiazole) in RBC gate was represented by histogram. First, it was represented isotype control and subsequently an example of reticulocyte labeling.


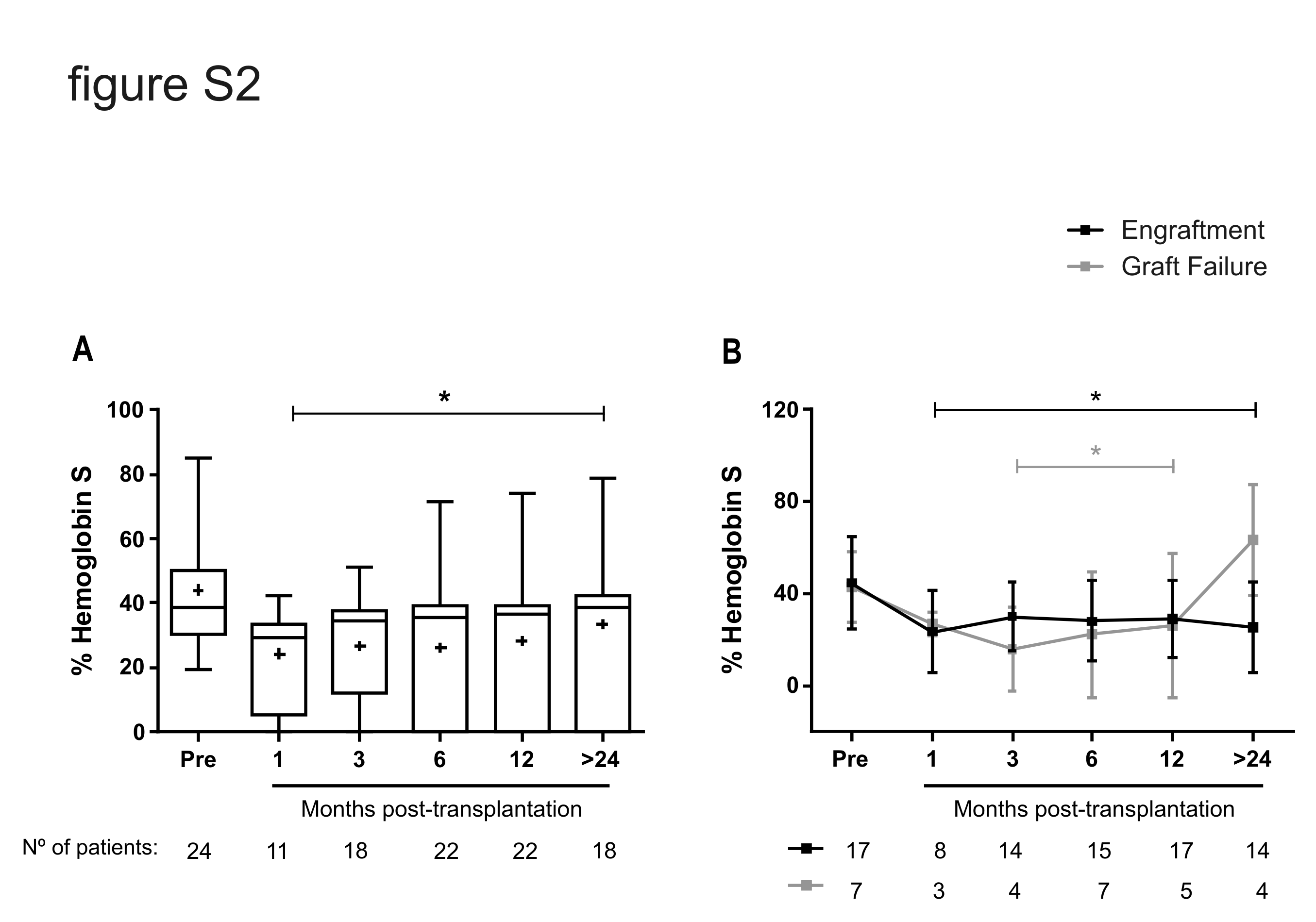


**Figure S2 – Hemoglobin S level in SCD patients following allogeneic HSCT.** Percentage of hemoglobin S (A) in the overall group of transplanted patients and (B) in patients divided according to graft function. Black line representing the engraftment group and gray line representing the graft failure group, + indicate the means. Statistical analysis was performed using a model of multiple regression of mixed effects. *Statistical difference between pre- and post-transplantation time points in the overall group of patients (A) or in each group (B) (P <0.05); **#**Statistical difference between engraftment group and graft failure group (P <0.05); Pre: pre-transplant period.

**
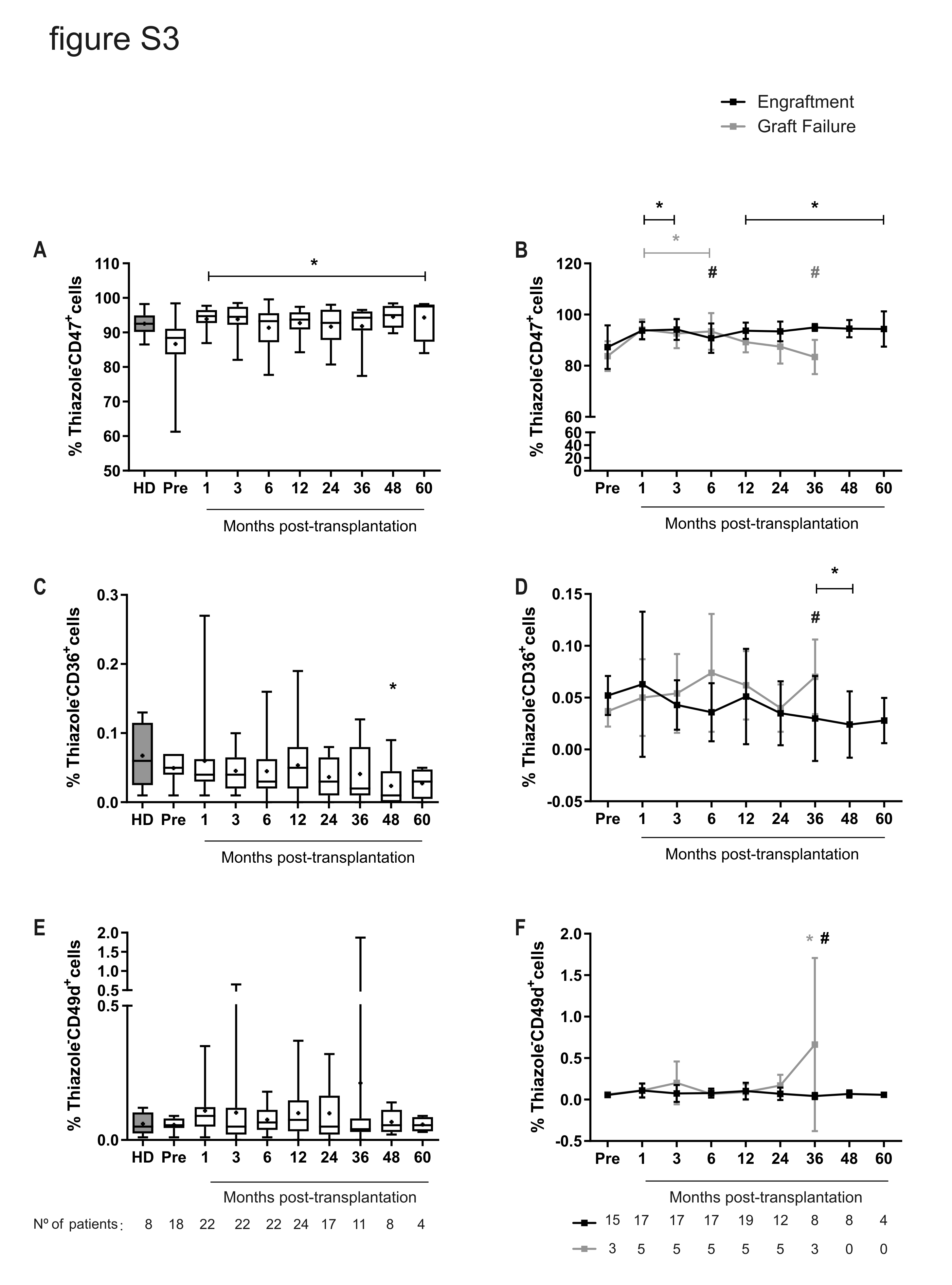
**

**Figure S3 - Expression of adhesion molecules in mature RBC from SCD-patients treated with allogeneic HSCT.** (A) percentage of mature RBC expressing CD47 in the overall group of transplanted patients, (B) percentage of mature RBC expressing CD47 in patients divided according to graft function, (C) percentage of mature RBC expressing CD36 in transplanted patients, (D) percentage of mature RBC expressing CD36 in patients divided according to graft function, (E) percentage of mature RBC expressing CD49d in transplanted patients and (F) percentage of mature RBC expressing CD49d in patients divided according to graft function. Black line representing the engraftment group and gray line representing the graft failure group, + indicate the means. Statistical analysis was performed using a model of multiple regression of mixed effects. *****Statistical difference between pre- and post-transplantation time points in the overall group of patients (A,C,E) or in each group (B,D,F) (P <0.05); ** Statistical difference between healthy donors and pre-transplantation (P <0.05). **#**Statistical difference between engraftment group and graft failure group (P <0.05); HD: healthy donor; Pre: pre-transplantation period.

**
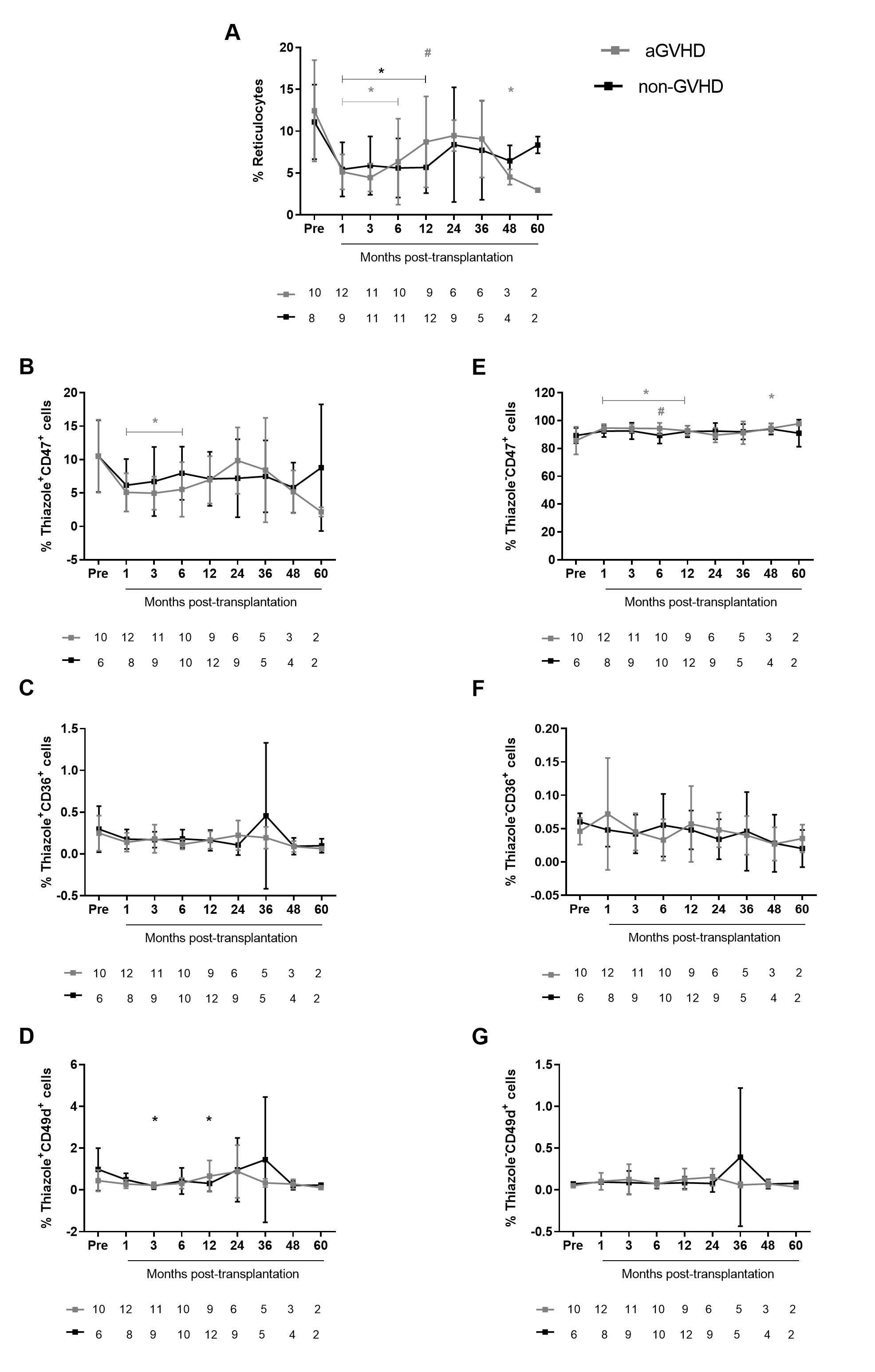
**

**Figure S4 - Expression of adhesion molecules in reticulocytes and mature RBC from SCD-patients treated with allogeneic HSCT.** (A) reticulocytes in patients divided according to GVHD (B) percentage of reticulocytes expressing CD47 in patients divided according to GVHD, (C) percentage of reticulocytes expressing CD36 in patients divided according to GVHD, (D) percentage of reticulocytes expressing CD49d in patients divided according to GVHD, (E) percentage of mature RBC expressing CD47 in patients divided according to GVHD, (F) percentage of mature RBC expressing CD36 in patients divided according to GVHD (G) percentage of mature RBC expressing CD49d in patients divided according to GVHD. Black line representing the non-GVHD group and gray line representing the aGVHD group, + indicate the means. Statistical analysis was performed using a model of multiple regression of mixed effects. *****Statistical difference between pre- and post-transplantation time points in each group (P <0.05); **#**Statistical difference between aGVHD group and non-GVHD group (P <0.05); Pre: pre-transplantation period.

**
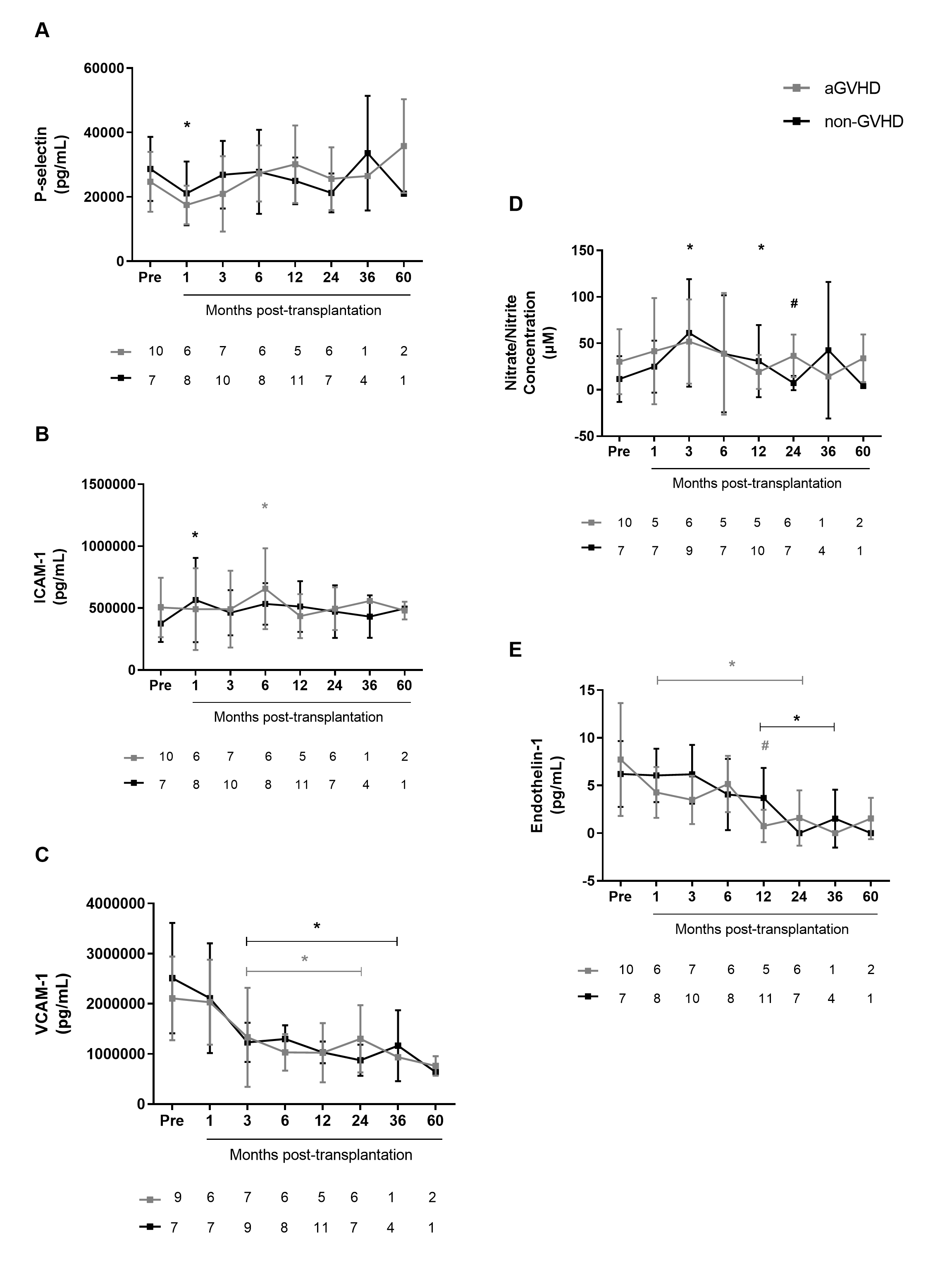
**

**Figure S5 - Levels of soluble adhesion molecules** **and markers of hemolysis and muscular tone in SCD patients following allogeneic HSCT.** (A) P-selectin in patients divided according to GVHD (B) ICAM-1 in patients divided according to GVHD, (C) VCAM-1 in patients divided according to GVHD, (D) nitrite and nitrate in patients divided according to GVHD, (E) endothelin-1 in patients divided according to GVHD. Black line representing the non-GVHD group and gray line representing the aGVHD group, + indicate the means. Statistical analysis was performed using a model of multiple regression of mixed effects. *****Statistical difference between pre- and post-transplantation time points in each group (P <0.05); **#**Statistical difference between aGVHD group and non-GVHD group (P <0.05); Pre: pre-transplantation period.

**
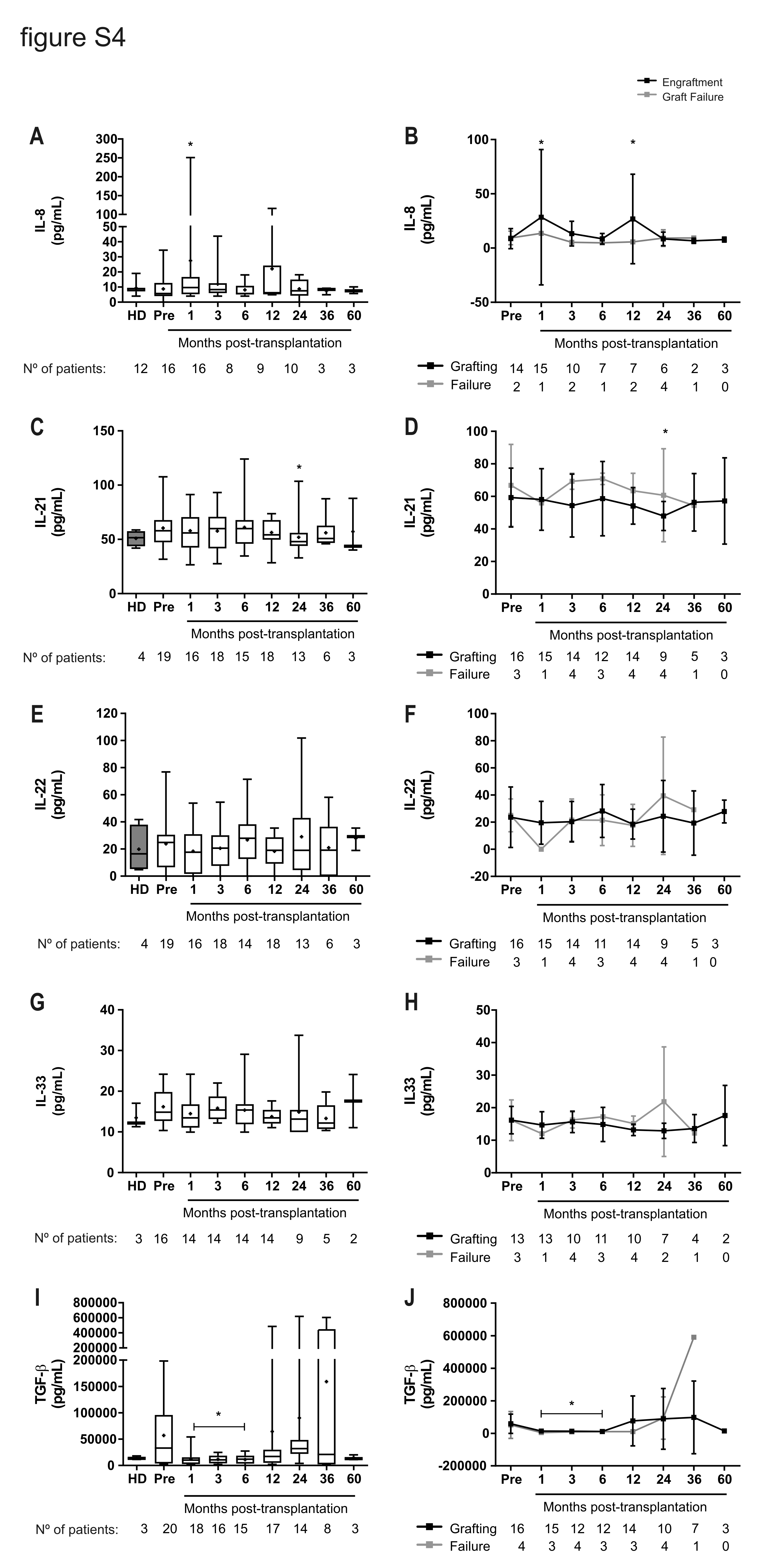
**

**Figure S6 - Levels of inflammatory mediators in SCD patients following allogeneic HSCT.** Concentration of (A) IL-8 in the overall group of transplanted patients, (B) IL-8 in patients divided according to graft function, (C) IL-21 in the overall group of transplanted patients, (D) IL-21 in patients divided according to graft function, (E) IL-22 in the overall group of transplanted patients, (F) IL-22 in patients divided according to graft function, (G) IL-33 in the overall group of transplanted patients, (H) IL-33 in patients divided according to graft function, (J) TGF-β in the overall group of transplanted patients, (I) TGF-β in patients divided according to graft function. Black line representing the engraftment group and gray line representing the graft failure group, + indicate the means. Statistical analysis was performed using a model of multiple regression of mixed effects. *****Statistical difference between pre- and post-transplantation time points in the overall group of patients (A,C,E,G,I) or in each group (B,D,F,H,J) (P <0.05); ** Statistical difference between healthy donors and pre-transplantation (P <0.05). **#**Statistical difference between engraftment group and graft failure group (P <0.05); HD: healthy donor; Pre: pre-transplantation period.

**
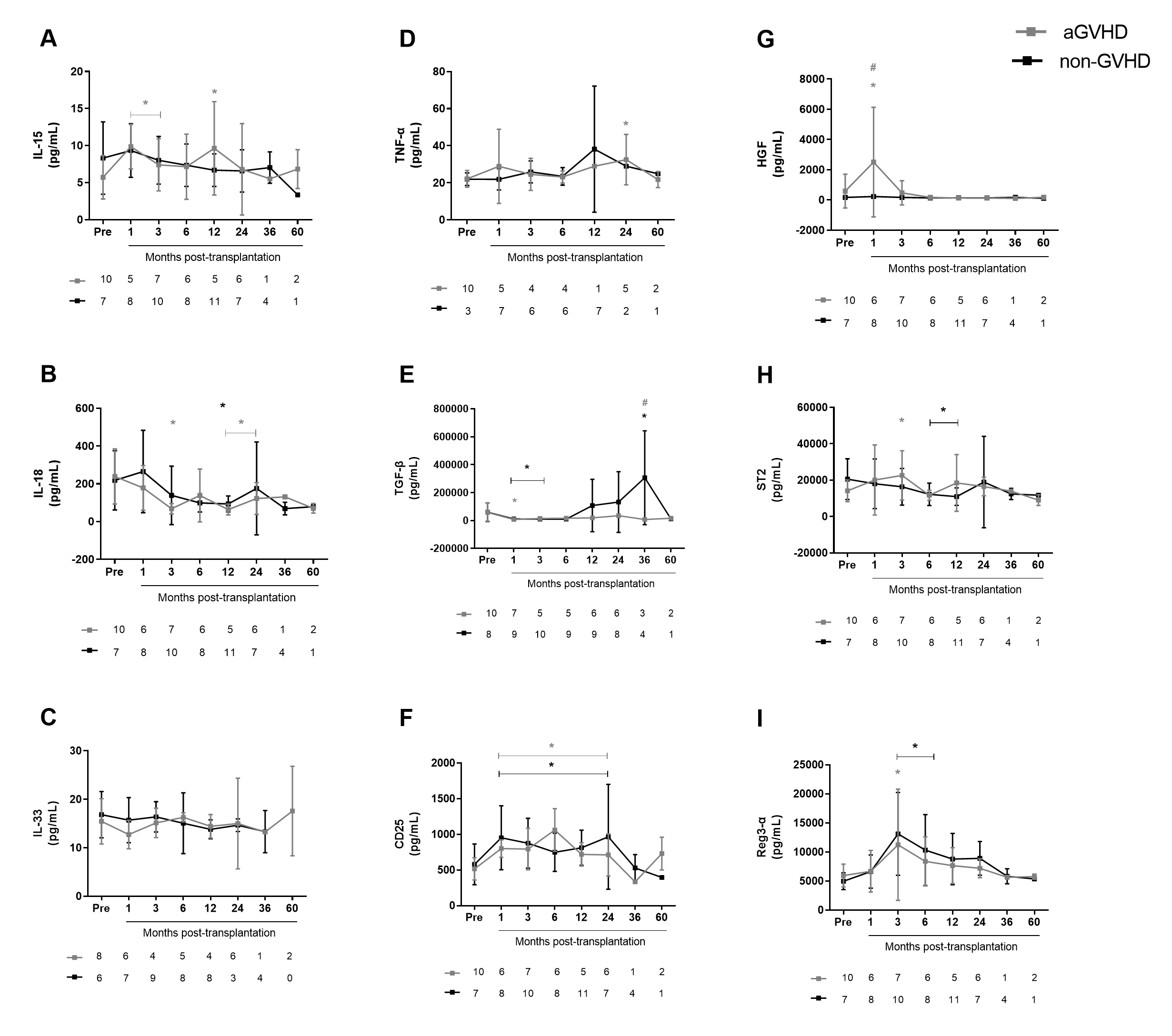
**

**Figure S7 - Levels of inflammatory mediators in SCD patients following allogeneic HSCT.** (A) IL-15 in patients divided according to GVHD (B) IL-18 in patients divided according to GVHD, (C) IL-33 in patients divided according to GVHD, (D) TNF-a in patients divided according to GVHD, (E) TGF-b in patients divided according to GVHD, (F) CD25 in patients divided according to GVHD, (G) HGF in patients divided according to GVHD, (H) ST2 in patients divided according to GVHD, (I) Reg3-a in patients divided according to GVHD. Black line representing the non-GVHD group and gray line representing the aGVHD group, + indicate the means. Statistical analysis was performed using a model of multiple regression of mixed effects. *****Statistical difference between pre- and post-transplantation time points in each group (P <0.05); **#**Statistical difference between aGVHD group and non-GVHD group (P <0.05); Pre: pre-transplantation period.

**Supplementary Tables**

**Table S1 - Patient characteristics and clinical outcomes of 32 SCD patients treated with allogeneic HSCT**

| **Patient N.** | **Genotype** | **Sex/Age (y) at baseline** | **Race** | **Baseline treatment** | **Donor** | **Conditioning Regimen** | **Transplant date** | **Follow-up**  **(months)** | **aGVHD** | **cGVHD** | **Graft function** | **Death** |
| --- | --- | --- | --- | --- | --- | --- | --- | --- | --- | --- | --- | --- |
| **SCD01** | HbSS | F/9 | White | Chronic transfusion | Sickle cell trait | Myeloablative  (BUCY-ATG) | 04/Oct/2010 | 45 | Grade I (GIT)  (not available*) | No | Engraftment | No |
| **SCD02** | HbSS | M/20 | Biracial | Hydroxyurea | Sickle cell trait negative | Myeloablative  (BUFLU-ATG) | 15/Oct/2010 | 44 | Grade I (skin)  (not available*) | No | Engraftment | No |
| **SCD03** | HbS/β0 | F/27 | White | Hydroxyurea | Sickle cell trait | Myeloablative  (BUFLU-ATG) | 19/Jul/2011 | 37 | No | No | Engraftment | No |
| **SCD04** | HbSS | F/17 | White | Hydroxyurea and Chronic transfusion | Sickle cell trait negative | Myeloablative  (BUFLU-ATG) | 21/Jun/2012 | 38 | No | No | Engraftment | No |
| **SCD05** | HbSS | F/14 | White | Hydroxyurea | Sickle cell trait negative | Myeloablative  (BUFLU-ATG) | 05/Feb/2013 | 42 | No | Mild (skin)  (10 mo after HSCT) | Engraftment | No |
| **SCD06** | HbSS | F/10 | White | Chronic transfusion | Sickle cell trait | Myeloablative  (BUFLU-ATG) | 15/Mar/2013 | 36 | No | No | Engraftment | No |
| **SCD07** | HbS/β0 | M/35 | Biracial | Chronic transfusion | Sickle cell trait | Myeloablative  (BUFLU-ATG) | 12/Apr/2013 | 52 | Grade II (skin)  (6 mo after HSCT) | No | Engraftment | No |
| **SCD08** | HbS/β0 | F/31 | White | Hydroxyurea | Sickle cell trait | Myeloablative  (BUFLU-ATG) | 02/Jul/2013 | 49 | No | No | Poor graft Function  (not available*) | Yes |
| **SCD09** | HbSS | M/24 | Biracial | Hydroxyurea and Chronic transfusion | Sickle cell trait negative | Myeloablative  (BUFLU-ATG) | 06/Aug/2013 | 48 | No | No | Engraftment | No |
| **SCD10** | HbS/β0 | M/13 | White | Chronic transfusion | Sickle cell trait negative | Myeloablative  (BUFLU-ATG) | 27/Nov/2013 | 45 | No | No | Failure  (1 yr and 2 mo after HSCT) | No |
| **SCD11** | HbS/β0 | F/30 | White | Chronic transfusion | Sickle cell trait | Myeloablative  (BUFLU-ATG) | 05/Feb/2014 | 42 | No | No | Engraftment | No |
| **SCD12** | HbS/β0 | M/25 | Biracial | Hydroxyurea | Sickle cell trait negative | Myeloablative  (BUFLU-ATG) | 19/Mar/2014 | 41 | Grade II (skin)  (10 mo after HSCT) | Moderate (skin)  (1 mo after HSCT) | Failure  (1 yr and 3 mo after HSCT) | No |
| **SCD13** | HbS/HbC | M/7 | Biracial | Hydroxyurea and Chronic transfusion | Sickle cell trait | Myeloablative  (BUFLU-ATG) | 24/Jul/2014 | 37 | Grade I (skin)  (1 mo after HSCT) | Moderate (skin)  (11 mo after HSCT) | Engraftment | No |
| **SCD14** | HbSS | M/18 | White | Hydroxyurea and Chronic transfusion | Sickle cell trait | Myeloablative  (BUFLU-ATG) | 10/Jul/2014 | 37 | Grade III (skin)  (2 mo after HSCT) | No | Engraftment | No |
| **SCD15** | HbSS | F/14 | White | Hydroxyurea | Sickle cell trait | Myeloablative  (BUFLU-ATG) | 03/Sep/2014 | 35 | Grade II (skin)  (1.5 mo after HSCT) | No | Failure  (10 mo after HSCT) | No |
| **SCD16** | HbSS | F/23 | Biracial | Chronic transfusion | Sickle cell trait negative | Myeloablative  (BUFLU-ATG) | 24/Sep/2014 | 35 | No | No | Engraftment | No |
| **SCD17** | HbSS | F/19 | White | Hydroxyurea | Sickle cell trait | Myeloablative  (BUFLU-ATG) | 02/Dec/2014 | 32 | No | No | Engraftment | No |
| **SCD18** | HbSS | M/14 | Biracial | Hydroxyurea and Chronic transfusion | Sickle cell trait negative | Myeloablative  (BUFLU-ATG) | 13/Nov/2014 | 33 | Grade I (skin)  (9 mo after HSCT) | No | Engraftment | No |
| **SCD19** | HbSS | F/10 | White | Chronic transfusion | Sickle cell trait | Myeloablative  (BUFLU-ATG) | 30/Jan/2015 | 31 | No | No | Engraftment | No |
| **SCD20** | HbSS | M/11 | Biracial | Hydroxyurea and Chronic transfusion | Sickle cell trait | Myeloablative  (BUFLU-ATG) | 01/Apr/2015 | 28 | No | Mild (skin)  (2 mo after HSCT) | Engraftment | No |
| **SCD21** | HbSS | M/10 | White | Chronic transfusion | Sickle cell trait negative | Myeloablative  (BUFLU-ATG) | 08/Jul/2015 | 25 | No | No | Failure  (1 yr and 11 mo after HSCT) | No |
| **SCD22** | HbSS | M/30 | Biracial | * | Sickle cell trait | Myeloablative  (BUFLU-ATG) | 11/Dec/2015 | 20 | Grade II (skin)  (1 mo after HSCT) | No | Engraftment | No |
| **SCD23** | HbS/HbC | M/26 | White | Hydroxyurea | Sickle cell trait | Myeloablative  (BUFLU-ATG) | 25/Nov/2015 | 21 | No | No | Engraftment | No |
| **SCD24** | HbS/β0 | M/16 | White | Hydroxyurea | Sickle cell trait negative | Myeloablative  (BUFLU-ATG) | 04/Dec/2015 | 20 | No | No | Engraftment | No |
| **SCD25** | HbSS | M/12 | White | Chronic transfusion | Sickle cell trait | Myeloablative  (BUFLU-ATG) | 15/Mar/2016 | 17 | Grade I (skin)  (not available*) | No | Engraftment | No |
| **SCD26** | HbSS | M/20 | Biracial | Hydroxyurea | Sickle cell trait | Myeloablative  (BUFLU-ATG) | 06/Apr/2016 | 16 | No | No | Engraftment | No |
| **SCD27** | HbSS | M/7 | White | Chronic transfusion | Sickle cell trait | Myeloablative  (BUFLU-ATG) | 06/Jul/2016 | 13 | No | Mild (skin)  (1yr and 4mo after HSCT) | Engraftment | No |
| **SCD28** | HbSS | F/33 | Black | Hydroxyurea | Sickle cell trait | Myeloablative  (BUFLU-ATG) | 13/Jul/2016 | 13 | Grade II (GIT)  (2 mo after HSCT) | No | Engraftment | No |
| **SCD29** | HbSS | M/12 | White | Hydroxyurea | Sickle cell trait | Myeloablative  (BUFLU-ATG) | 10/Nov/2016 | 9 | Grade II (skin)  (1 yr and 4 mo after HSCT) | No | Engraftment | No |
| **SCD30** | HbSS | F/32 | White | Hydroxyurea | Sickle cell trait negative | Myeloablative  (BUFLU-ATG) | 19/Jan/2017 | 7 | Grade II (skin)  (1 mo after HSCT) | No | Failure  (5 mo after HSCT) | Yes |
| **SCD31** | HbSS | F/13 | White | Simple and Chronic transfusion | Sickle cell trait | Myeloablative  (BUFLU-ATG) | 09/Feb/2017 | 6 | Grade II (skin)  (15 days after HSCT) | No | Failure  (5 mo after HSCT) | No |
| **SCD32** | HbSS | F/12 | Biracial | Hydroxyurea and Chronic transfusion | Sickle cell trait | Myeloablative  (BUFLU-ATG) | 12/Apr/2017 | 4 | Grade II (skin)  (1 mo after HSCT) | Moderate (skin)  (6 mo after HSCT) | Engraftment | No |

*Data not available in medical charts; Myeloablative conditioning regimen: busulfan (12-16/mg/kg) and fludarabine (120mg/kg) plus rabbit anti-thymocyte globulin (4.5 mg) (BUFLU-ATG), or busulfan (12-16/mg/kg) and cyclophosphamide (200mg/Kg) plus rabbit anti-thymocyte globulin (4.5 mg) (BUCY-ATG). GIT: gastrointestinal tract.

GVHD first line treatment was initiated at diagnosis and consisted of corticosteroids (1mg/kg/day in moderate/severe chronic GVHD, and 2mg/kg/day in acute GVHD grade 2-4).

**Table S2 - Patients retrospectively clustered in graft failure and engraftment groups**

| **Patient N.** | **Genotype** | **Gender** | **Age (y) at baseline** | **Race** | **# Myeloablative Conditioning Regimen intensity** |
| --- | --- | --- | --- | --- | --- |
| **Graft Failure Group** | | | | | |
| **SCD08** | HbS/β0 | F | 31 | White | BUFLU-ATG |
| **SCD10** | HbS/β0 | M | 13 | White | BUFLU-ATG |
| **SCD12** | HbS/β0 | M | 24 | Biracial | BUFLU-ATG |
| **SCD15** | HbSS | F | 14 | White | BUFLU-ATG |
| **SCD21** | HbSS | M | 10 | White | BUFLU-ATG |
| **SCD30** | HbSS | F | 32 | White | BUFLU-ATG |
| **SC31** | HbSS | F | 13 | White | BUFLU-ATG |
| **Engraftment Group** | | | | | |
| **SCD01** | HbSS | F | 9 | White | BUCY-ATG |
| **SCD02** | HbSS | M | 20 | Biracial | BUFLU-ATG |
| **SCD03** | HbS/β0 | F | 27 | White | BUFLU-ATG |
| **SCD04** | HbSS | F | 17 | White | BUFLU-ATG |
| **SCD05** | HbSS | F | 14 | White | BUFLU-ATG |
| **SCD06** | HbSS | F | 10 | White | BUFLU-ATG |
| **SCD07** | HbS/β0 | M | 35 | Biracial | BUFLU-ATG |
| **SCD09** | HbSS | M | 24 | Biracial | BUFLU-ATG |
| **SCD11** | HbS/β0 | F | 30 | White | BUFLU-ATG |
| **SCD13** | HbS/HbC | M | 7 | Biracial | BUFLU-ATG |
| **SCD14** | HbSS | M | 18 | White | BUFLU-ATG |
| **SCD16** | HbSS | F | 23 | Biracial | BUFLU-ATG |
| **SCD17** | HbSS | F | 19 | White | BUFLU-ATG |
| **SCD18** | HbSS | M | 14 | Biracial | BUFLU-ATG |
| **SCD19** | HbSS | F | 10 | White | BUFLU-ATG |
| **SCD20** | HbSS | M | 11 | Biracial | BUFLU-ATG |
| **SCD22** | HbSS | M | 30 | Biracial | BUFLU-ATG |
| **SCD23** | HbS/HbC | M | 26 | White | BUFLU-ATG |
| **SCD24** | HbS/β0 | M | 16 | White | BUFLU-ATG |
| **SCD25** | HbSS | M | 12 | White | BUFLU-ATG |
| **SCD26** | HbSS | M | 20 | Biracial | BUFLU-ATG |
| **SCD27** | HbSS | M | 7 | White | BUFLU-ATG |
| **SCD28** | HbSS | F | 33 | Black | BUFLU-ATG |
| **SCD29** | HbSS | M | 12 | White | BUFLU-ATG |
| **SCD32** | HbSS | F | 12 | Biracial | BUFLU-ATG |

^#^Myeloablative conditioning regimen: busulfan (12-16/mg/kg) and fludarabine (120mg/kg) plus rabbit anti-thymocyte globulin (4.5 mg) (BUFLU-ATG), or busulfan (12-16/mg/kg) and cyclophosphamide (200mg/Kg) plus rabbit anti-thymocyte globulin (4.5 mg) (BUCY-ATG).

**Table S3 - Qualitative chimerism of SCD patients after allogeneic HSCT**

| **Patient Nº** | **1 mo** | **3 mo** | **6 mo** | **12 mo** | **24 mo** | **36 mo** | **48 mo** | **60 mo** |
| --- | --- | --- | --- | --- | --- | --- | --- | --- |
| **SCD01** |  |  |  |  |  |  | mixed | Mixed |
| **SCD02** |  |  |  |  |  | mixed | mixed |  |
| **SCD03** | donor | donor |  | donor | donor | donor | donor | Donor |
| **SCD04** | mixed | mixed | mixed | mixed | mixed | mixed | mixed | Mixed |
| **SCD05** |  | mixed | mixed | donor | mixed | donor | donor |  |
| **SCD06** | mixed |  | donor | donor | donor | donor |  |  |
| **SCD07** |  |  | mixed | mixed | mixed | mixed | mixed |  |
| **SCD08** |  | donor | mixed | mixed | mixed | Φ |  |  |
| **SCD09** | mixed | mixed | mixed | mixed | mixed | mixed | mixed |  |
| **SCD10** | mixed | mixed | mixed | mixed | **recipient** | **recipient** |  |  |
| **SCD11** | donor | donor | donor | donor | donor | donor |  |  |
| **SCD12** | mixed | mixed | mixed | mixed |  | **recipient** |  |  |
| **SCD13** | mixed | mixed | mixed | mixed | mixed |  |  |  |
| **SCD14** | mixed | mixed | mixed | mixed | donor | donor |  |  |
| **SCD15** |  | mixed | mixed | mixed | **recipient** |  |  |  |
| **SCD16** | mixed | mixed | mixed | mixed | mixed |  |  |  |
| **SCD17** | mixed | mixed | mixed | mixed | mixed |  |  |  |
| **SCD18** | mixed | mixed | mixed | mixed |  |  |  |  |
| **SCD19** | mixed | mixed | mixed | mixed | mixed |  |  |  |
| **SCD20** | mixed | mixed | mixed | mixed | mixed |  |  |  |
| **SCD21** | mixed | mixed | mixed | mixed | **recipient** |  |  |  |
| **SCD22** | mixed | mixed | mixed | mixed |  |  |  |  |
| **SCD23** |  | mixed | mixed | mixed |  |  |  |  |
| **SCD24** | mixed | mixed | mixed | mixed |  |  |  |  |
| **SCD25** | mixed | mixed | mixed | mixed |  |  |  |  |
| **SCD26** | mixed | mixed | mixed | mixed |  |  |  |  |
| **SCD27** | mixed | mixed | mixed | mixed |  |  |  |  |
| **SCD28** | donor | donor | donor | donor |  |  |  |  |
| **SCD29** | mixed |  | mixed |  |  |  |  |  |
| **SCD30** | donor | # |  |  |  |  |  |  |
| **SCD31** | mixed | mixed | mixed | Φ |  |  |  |  |
| **SCD32** | mixed |  |  |  |  |  |  |  |

Φ Temporary aplasia without transport support, increased levels of HbS (66%), with mixed chimera by the qualitative VNTR assay

# Bone marrow aplasia, with transfusion support, with mixed chimera by the qualitative VNTR assay

**Table S4 - Intergroup statistical analyses regarding age, gender and race of retrospectively divided groups**

| **Graft failure vs Engraftment groups** | | | |
| --- | --- | --- | --- |
| Age mean (SD) | Good graft  Graft failure | 18.50(8.39)  19.57(9.25) | p= 0.7726 (ns) |
| Female/male | Good graft  Graft failure | 11/14  4/3 | p= 0.6783 (ns) |
| White/non-white | Good graft  Graft failure | 14/11  6/1 | p= 0.2117 (ns) |

**Table S5 - Soluble mediators of inflammatory response in SCD patients submitted to allogeneic HSCT**

|  |  | **HD** | **Pre-Tx** | **1 mo** | **3 mo** | **6 mo** | **12 mo** | **24 mo** | **36 mo** | **60 mo** |
| --- | --- | --- | --- | --- | --- | --- | --- | --- | --- | --- |
| **IL1-β** | Number | 4 | 14 | 15 | 17 | 13 | 16 | 12 | 6 | 1 |
|  | Mean | 0 | 0 | 0 | 0 | 0 | 0.2025 | 0.1967 | 0 | 0 |
|  | SD | 0 | 0 | 0 | 0 | 0 | 0.81 | 0.4697 | 0 | 0 |
| **IL-2** | Number | 4 | 13 | 11 | 15 | 13 | 16 | 8 | 4 | 2 |
|  | Mean | 0 | 0 | 0 | 0 | 0 | 1.431 | 1.063 | 0 | 0 |
|  | SD | 0 | 0 | 0 | 0 | 0 | 3.92 | 3.005 | 0 | 0 |
| **IL-4** | Number | 4 | 15 | 16 | 17 | 12 | 17 | 10 | 4 | 2 |
|  | Mean | 0 | 0.04133 | 0.1775 | 0.15 | 0 | 0.8335 | 0.673 | 0 | 0.42 |
|  | SD | 0 | 0.1601 | 0.71 | 0.6185 | 0 | 2.613 | 1.202 | 0 | 0.594 |
| **IL-5** | Number | 3 | 13 | 12 | 15 | 10 | 17 | 7 | 4 |  |
|  | Mean | 0 | 0 | 0 | 0 | 1.664 | 15.93 | 3.643 | 0 |  |
|  | SD | 0 | 0 | 0 | 0 | 5.262 | 47.16 | 9.638 | 0 |  |
| **IL-7** | Number | 4 | 19 | 16 | 18 | 14 | 18 | 12 | 6 | 3 |
|  | Mean | 0 | 4.763 | 12.72 | 7.598 | 20.6 | 46.33 | 6.87 | 5.562 | 4.543 |
|  | SD | 0 | 12.25 | 29.97 | 16.76 | 61.73 | 193.6 | 11.01 | 9.997 | 7.869 |
| **IL-10** | Number | 3 | 6 | 5 | 13 | 6 | 13 | 6 | 1 |  |
|  | Mean | 0 | 1.673 | 3.872 | 0.6562 | 45.19 | 3.558 | 3.362 | 0 |  |
|  | SD | 0 | 4.099 | 5.623 | 2.366 | 110.7 | 10.89 | 5.41 | 0 |  |
| **IL-12** | Number | 3 | 13 | 11 | 14 | 10 | 16 | 5 | 2 |  |
|  | Mean | 0 | 0 | 0 | 0.4943 | 17.58 | 16.73 | 2.394 | 0 |  |
|  | SD | 0 | 0 | 0 | 1.849 | 51.17 | 56.6 | 5.353 | 0 |  |
| **IL-13** | Number | 4 | 18 | 16 | 18 | 14 | 18 | 10 | 5 | 2 |
|  | Mean | 0 | 0 | 0.18 | 0.16 | 27.27 | 0.7256 | 1.638 | 0 | 0 |
|  | SD | 0 | 0 | 0.72 | 0.6788 | 102 | 1.804 | 3.085 | 0 | 0 |
| **IL-17** | Number | 3 | 12 | 15 | 15 | 10 | 14 | 7 | 2 |  |
|  | Mean | 0 | 0 | 0.844 | 0.844 | 0 | 3.733 | 1.994 | 0 |  |
|  | SD | 0 | 0 | 3.269 | 3.269 | 0 | 10.03 | 5.276 | 0 |  |
| **G-CSF** | Number | 3 | 17 | 14 | 17 | 13 | 17 | 12 | 6 | 3 |
|  | Mean | 0 | 22.02 | 117.8 | 2.161 | 50.08 | 0 | 4207 | 0 | 0 |
|  | SD | 0 | 44.9 | 440.9 | 8.908 | 180.6 | 0 | 14574 | 0 | 0 |
| **GM-CSF** | Number | 3 | 18 | 14 | 17 | 13 | 18 | 12 | 5 | 2 |
|  | Mean | 0 | 0 | 0 | 0 | 0 | 1.794 | 0.6375 | 0 | 0 |
|  | SD | 0 | 0 | 0 | 0 | 0 | 4.544 | 1.653 | 0 | 0 |

**Table S6 – ROC curves analysis of endothelin-1 levels before transplantation**

| **Candidate biomarker** | | **Period post-HSCT** | | **Group** | **n** | **AUC** | **CI 95%** | |
| --- | --- | --- | --- | --- | --- | --- | --- | --- |
| Endothelin-1 | | Pre-tx | | Graf failure | 3 | 0.66 | 0.41-0.85 | |
|  |  |  |  | Good graft function | 16 |  |  |  |
|  | | | | | | | | |
| **Cut-off** | **Sensitivity (%)** | **CI 95%** | **Specificity (%)** | **CI 95%** | **PPV** | **CI 95%** | **NPV** | **CI 95%** |
| ≥0 | 100 | 29.2 - 100.0 | 0 | 0.0 - 20.6 | 15.8 | 3.4 - 39.6 |  |  |
| >0 | 100 | 29.2 - 100.0 | 12.5 | 1.6 - 38.3 | 17.6 | 3.8 - 43.4 | 100 | 15.8 - 100.0 |
| >1.33 | 66.67 | 9.4 - 99.2 | 12.5 | 1.6 - 38.3 | 12.5 | 1.6 - 38.3 | 66.7 | 9.4 - 99.2 |
| >3.07 | 66.67 | 9.4 - 99.2 | 18.75 | 4.0 - 45.6 | 13.3 | 1.7 - 40.5 | 75 | 19.4 - 99.4 |
| >5.03 | 66.67 | 9.4 - 99.2 | 25 | 7.3 - 52.4 | 14.3 | 1.8 - 42.8 | 80 | 28.4 - 99.5 |
| >6.07 | 66.67 | 9.4 - 99.2 | 68.75 | 41.3 - 89.0 | 28.6 | 3.7 - 71.0 | 91.7 | 61.5 - 99.8 |
| >7.21 | 66.67 | 9.4 - 99.2 | 75 | 47.6 - 92.7 | 33.3 | 4.3 - 77.7 | 92.3 | 64.0 - 99.8 |
| >7.83 | 66.67 | 9.4 - 99.2 | 81.25 | 54.4 - 96.0 | 40 | 5.3 - 85.3 | 92.9 | 66.1 - 99.8 |
| >9.34 | 33.33 | 0.8 - 90.6 | 87.5 | 61.7 - 98.4 | 33.3 | 0.8 - 90.6 | 87.5 | 61.7 - 98.4 |
| >10.89 | 33.33 | 0.8 - 90.6 | 93.75 | 69.8 - 99.8 | 50 | 1.3 - 98.7 | 88.2 | 63.6 - 98.5 |
| >11.93 | 33.33 | 0.8 - 90.6 | 100 | 79.4 - 100.0 | 100 | 2.5 - 100.0 | 88.9 | 65.3 - 98.6 |
| >21.38 | 0 | 0.0 - 70.8 | 100 | 79.4 - 100.0 |  |  | 84.2 | 60.4 - 96.6 |

HSCT: hematopoietic stem-cell transplantation; AUC: area under the curve; CI: Confidence interval; PPV: Positive predictive value; NPV: Negative predictive value.

**Please see additional Excel Files**

(Results of the Statistical analyses)
